# Action Sounds Informing Own Body Perception Influence Gender Identity and Social Cognition

**DOI:** 10.3389/fnhum.2021.688170

**Published:** 2021-07-28

**Authors:** Sünje Clausen, Ana Tajadura-Jiménez, Christian P. Janssen, Nadia Bianchi-Berthouze

**Affiliations:** ^1^UCL Interaction Centre (UCLIC), University College London, London, United Kingdom; ^2^DEI Interactive Systems Group, Department of Computer Science and Engineering, Universidad Carlos III de Madrid (UC3M), Madrid, Spain; ^3^Experimental Psychology, Helmholtz Institute, Utrecht University, Utrecht, Netherlands; ^4^Research Group Digital Communication and Transformation, Department of Computer Science and Applied Cognitive Science, University of Duisburg-Essen, Duisburg, Germany

**Keywords:** body representation, body perception, multisensory perception, sound, own body illusion, self-concept, gender identity, implicit association test (IAT)

## Abstract

Sensory information can temporarily affect mental body representations. For example, in Virtual Reality (VR), visually swapping into a body with another sex can temporarily alter perceived gender identity. Outside of VR, real-time auditory changes to walkers’ footstep sounds can affect perceived body weight and masculinity/femininity. Here, we investigate whether altered footstep sounds also impact gender identity and relation to gender groups. In two experiments, cisgender participants (26 females, 26 males) walked with headphones which played altered versions of their own footstep sounds that sounded more typically male or female. Baseline and post-intervention measures quantified gender identity [Implicit Association Test (IAT)], relation to gender groups [Inclusion of the Other-in-the-Self (IOS)], and perceived masculinity/femininity. Results show that females felt more feminine and closer to the group of women (IOS) directly after walking with feminine sounding footsteps. Similarly, males felt more feminine after walking with feminine sounding footsteps and associated themselves relatively stronger with “female” (IAT). The findings suggest that gender identity is temporarily malleable through auditory-induced own body illusions. Furthermore, they provide evidence for a connection between body perception and an abstract representation of the Self, supporting the theory that bodily illusions affect social cognition through changes in the self-concept.

## Introduction

The brain has a mental representation of the body (e.g., its size, shape, configuration) which is continuously updated through multimodal sensory information from body-environment interactions (Tsakiris, 2010; Ehrsson, 2012). By experimentally altering such sensory information, the mental body representation can be temporarily changed (Botvinick and Cohen, 1998). A suitable method for inducing *own body illusions*, during which the body is perceived differently from its physical state, is altering auditory information from body-environment interactions (Stanton and Spence, 2020). In several studies, auditory feedback has been used to alter body weight perception (Tajadura-Jiménez et al., 2015), body height perception (Tajadura-Jiménez et al., 2018), and perception of the properties of limbs (Senna et al., 2014; Tajadura-Jiménez et al., 2017b). In this paper, we investigate if alterations of auditory feedback can lead to changes in the self-concept and feelings of belonging to a group.

While audition is a newcomer to research in multisensory changes in body perception, other studies have manipulated visual feedback to induce *body swap illusions*, in which participants are perceptually “swapped” into another body, for example an avatar in Virtual Reality (VR). Crucially, the embodiment of an avatar with physical features of an outgroup relative to the participant (i.e., different ethnicity, gender, age) has been shown to affect implicit attitudes toward the embodied outgroup (Fini et al., 2013; Peck et al., 2013; Farmer et al., 2014).

To explain these effects of *body swap illusions* on social cognition, Maister et al. (2015) and Tsakiris (2017) recently hypothesised a connection between body perception and higher-level cognition. In particular, Maister et al. (2015) propose that the mental representation of “me” contains both a representation of the body as well as more abstract facets of the Self, such as attitudes, beliefs, and relations to social in- and outgroups. While several experiments have shown that body swap illusions can affect (implicit) attitudes toward an outgroup (see e.g., Banakou et al., 2013; Peck et al., 2013; Tajadura-Jiménez et al., 2017a), there is less research on how to facilitate the changes in the self-concept and identification with social groups. Banakou et al. (2013) and Tajadura-Jiménez et al. (2017a) found that embodying a child avatar in VR increased implicit associations between the Self and child-like attributes which provides evidence for a link between the self-concept and the embodied group after experiencing a body swap illusion. With respect to gender identity, Tacikowski et al. (2020a) found that body swap illusions with an avatar of a different sex cause a more balanced gender identification with male and female gender.

While these studies focus on a complete alteration of the body taking place by embodying another person’s body, a connection between direct changes of one’s own actual body and the Self remains to be investigated. Our research addresses this gap by focussing on subtle *own body illusions* induced through auditory feedback from body-environment interactions and investigates their effect on the self-concept and the feeling of belonging to a social group. The effects of direct alterations of one’s own actual body perception are particularly relevant because this perception (and the body itself) might naturally change throughout life. If one’s body perception is related to one’s self-concept and social cognition, then these could situationally differ depending on the current state and perception of one’s body.

In a recent study, Tajadura-Jiménez et al. (2019) had found that auditory information from footsteps did not only affect body size perception but also the perceived masculinity and femininity of the participants, hence, suggesting a change in the perception of the Self through a subtle own body illusion. Building on these earlier findings that footstep sounds can affect perceived masculinity and femininity (Tajadura-Jiménez et al., 2019), and that gender identity is temporarily malleable through body swap illusions (Tacikowski et al., 2020a), here, we investigate whether altered footstep sounds could induce a “gender illusion” during which participants identify more strongly with their respective gender outgroup. Thereby, we understand gender as “*the meanings ascribed to male and female social categories within a culture*” (Wood and Eagly, 2015, p. 461) and adopting those cultural meanings into one’s own personality results in one’s gender identity. Gender identity is a multifaceted concept evolving through a combination of biological, cognitive, and social factors (Wendy and Eagly, 2009) and appears to be closely linked to one’s body perception (Tacikowski et al., 2020a).

One theory for explaining a possible influence of direct changes of one’s own actual body perception on gender identity is the predictive coding account (Clark, 2013; Apps and Tsakiris, 2014). From this theoretical lens, a gender illusion would be expected to occur as follows: while walking, the brain constantly predicts the sensory input it will receive, including the well-known sound of one’s own footsteps. The frequency components in the footstep sounds of female and male walkers are distinct and people can distinguish genders based on these sounds (Li et al., 1991; Giordano et al., 2014). Low frequency footstep sounds are typically associated with a more masculine, heavy walker, and with wearing flat shoes, while high frequency footstep sounds are typically associated with a more feminine, light walker, and with wearing high heels (Li et al., 1991). For most female individuals, the brain is therefore expected to predict more high frequency footstep sounds during walking. However, if the frequency components of footstep sounds are altered experimentally to emphasise the lower frequency bands, the perceived walking sounds differ substantially from the predicted sounds. This mismatch between predicted high frequency sounds and heard low frequency sounds creates prediction errors. To resolve the conflict and to reduce the occurrence of prediction errors, the mentally represented body is hypothesised to update in a way to look relatively heavier and bigger in size, thus, creating the bodily illusion of being more like a stereotypical male and less like a stereotypical female (Apps and Tsakiris, 2014).

The hypothesised prediction error caused by bodily illusions, including footstep sound manipulations, are expected to then impact people’s self-perception. Specifically, we hypothesise that bodily illusions from footstep sounds will blur the boundaries between Self and other (Paladino et al., 2010), increase self-association with the embodied outgroup member (Maister et al., 2015), and change higher-level concepts of the Self (Tsakiris, 2017). Therefore, we expect changes in implicit self-gender associations and explicit self-gender group identification following the induced *own body gender illusion.*

To investigate this idea, we report two experiments which altered footstep sounds in real-time to resemble more feminine or masculine footsteps during walking. We tested how these sounds change participants’ self-concept and the relation to social groups for cisgender females (Experiment I) and cisgender males (Experiment II). The following three hypotheses were formulated.

**H1**: Altered footstep sounds will affect *body perception*, as quantified by changes in *bodily feelings* and *related motor behaviour*. Frequency components in footstep sounds are generally associated with the sex and weight of the walker (Li et al., 1991), and previous research has induced illusory changes of one’s own actual body (i.e., own body illusions) through altering footstep sounds (Tajadura-Jiménez et al., 2015, 2019). Therefore, it was expected that participants will feel lighter and more feminine after walking with high frequency step sounds compared to low frequency step sounds as it was found in Tajadura-Jiménez et al. (2019). The previously found interaction effects also suggest a connection between footstep sounds and perceived strength (Tajadura-Jiménez et al., 2019). As lower strength is stereotypically associated with females, we also hypothesised that participants will feel relatively weaker after walking with high frequency step sounds compared to low frequency step sounds. The bodily feelings of perceived body weight, masculinity/femininity, and strength might jointly or interactively be related to the multifaceted concept of gender identity and were thus all considered in this study. The second part of the hypothesis builds on two observations in the literature. First, altering body representations during bodily illusions can change *motor behaviour*. For example, arm reaching movements (Tajadura-Jiménez et al., 2016) and step size (Tajadura-Jiménez et al., 2018) are adjusted together with experienced changes in arm/leg size; leg acceleration and foot-ground contact time are adjusted together with experienced changes in perceived body weight (Tajadura-Jiménez et al., 2015, 2019). Second, masculine and feminine gait differ in lateral hip and chest sway (Mather and Murdoch, 1994). Given these observations, it was hypothesised that the bodily illusion could cause participants to adjust their walking behaviour to resemble masculine walking patterns more closely in the low frequency condition and feminine walking patterns more closely in the high frequency condition.

**H2**: Altered footstep sounds will affect implicit *self-gender associations*. Bodily illusions are expected to affect the self-concept by increasing associations of the Self with the embodied group (Banakou et al., 2013; Maister et al., 2015; Tsakiris, 2017). Therefore, it was expected that high frequency footstep sounds will enhance self-female associations and that low frequency step sounds will enhance self-male associations.

**H3**: Altered footstep sounds will affect explicit *self-gender group identification*. Bodily illusions can increase identification with the embodied group (Maister et al., 2015; Tsakiris, 2017). Therefore, it was expected that high frequency footstep sounds will increase identification of the Self with the group of women, and that low frequency footstep sounds will increase identification of the Self with the group of men.

## Experiment I: Altering Implicit Self-Gender Association and Explicit Self-Gender Group Identification of Women

### Method

#### Participants

26 cisgender women took part in the first experiment (*M* = 26.31 years, *SD* = 4.46 years). On average, they weighed 58.73 kg (*SD* = 9.71 kg) and were self-reportedly 164.6 cm (*SD* = 8.03 cm) tall. Body mass index (*M* = 21.65, *SD* = 3.06, Range = 17.51 – 29.39) was in the healthy range (18.5 – 24.9) according to the National Health Service in the UK^1^ for 19 of the participants. Eligibility criteria included no history of hearing problems and no (history of) eating disorders, as previous research showed that individuals with a (history of) eating disorder differ in their body perception and sensitivity to bodily illusions (Eshkevari et al., 2012, 2014). Participants were recruited through an online subject pool, flyers, the researcher’s social network, and by asking people on campus. Participants could choose to participate in a raffle for one of three £30 Amazon vouchers (20 participants), to recruit the experimenter for their own experiment (6 participants), or to receive one academic credit (0 participants).

Ethical approval was obtained by the UCL Research Ethics Committee (Project ID: UCLIC/1516/003/Staff). The study was performed according to institutional ethics and international standards for the protection of human participants. All participants provided written informed consent prior to participation and were fully debriefed.

#### Materials

**FIGURE 1:**
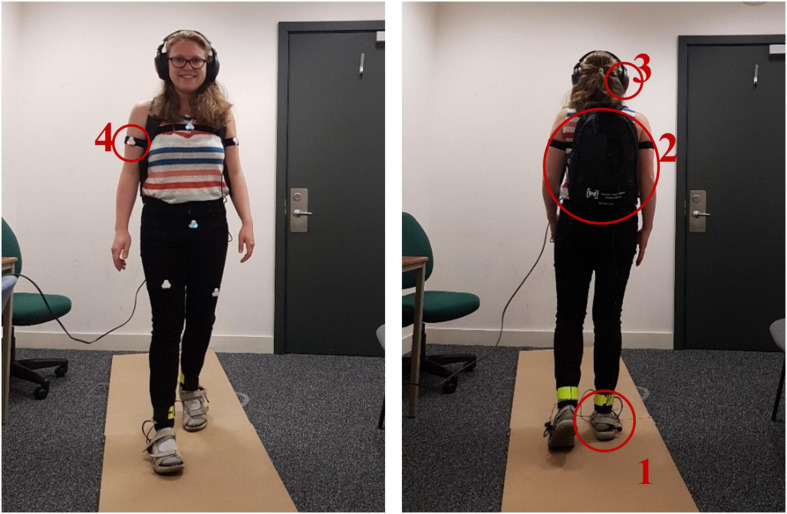
Equipment during the two walking phases. Participants wore strap-sandals with attached microphones (1), a small backpack containing a preamplifier and an equaliser (2), and headphones through which participants heard their altered footstep sounds (3). Black rubber bands with one of six Notch movement sensors (white triangles) each (4) were attached to the upper arms, chest, hip, and thighs.

Participants walked with two types of altered footstep sounds in one “walking phase” respectively. During these walking phases, participants wore a set of equipment in order to alter footstep sounds in real-time and to capture behavioural data. The full set-up is displayed in Figure 1. The experiment was conducted in a quiet room and participants walked on a 3.6 × 0.6 m wooden corridor (medium density fibre, 2.5 cm thick). Questionnaires and tasks were presented on a 14” laptop (Intel Core i7, 16 GB RAM).

##### Real-time sound alteration

The equipment for altering footstep sounds in real-time involved a pair of strap-sandals similar to the one used by Tajadura-Jiménez et al. (2015). These sandals had a hard rubber sole and produced clear contact sound on the wooden corridor during walking. In order to capture and consecutively alter the footstep sounds, a pair of small microphones (Core Sound) were attached to the sandals (one microphone on each). These microphones were connected to a preamplifier (FoneStar, TC-6M) to increase the loudness of the captured sounds. The preamplifier was connected to a stereo 9-band graphic equaliser (Behringer FBQ800) which allowed the enhancement or diminution of the loudness of certain frequency components in the sounds (see sound conditions in Experimental Design). During walking, the participants heard their altered footstep sounds through a pair of headphones (Sennheiser HDA 300) with high passive noise attenuation (>30 dBA) that muffled the actual sound of footsteps. The preamplifier and equaliser were fitted into a small backpack for the participant to carry (∼2 kg).

##### Measuring instruments

(a) *Bodily feelings and motor behaviour.* Analogous to previous work (Tajadura-Jiménez et al., 2019), bodily feelings were measured with three statements on a 7-point Likert scale: I felt… (1) “light” to “heavy,” (2) “weak” to “strong,” and (3) “very feminine” to “very masculine.” These self-report questions were used to measure whether the altered footstep sounds affected participants similarly as in previous research using altered footstep sounds, and hence, whether the bodily illusion was induced successfully. As perceived weight, femininity/masculinity, and strength all relate to the multifaceted concept of gender identity, we refer to this bodily illusion as a gender illusion. To measure typically masculine and feminine gait features (i.e., lateral hip and shoulder sway) each participant wore 6 Notch movement sensors^2^ attached with rubber bands to their upper arms, thighs, chest, and hips. The movement data was recorded with the Notch Pioneer Motion Capture application (v. 1.10.0) on an Android Samsung Galaxy S7 Smartphone.

(b) *Implicit self-gender associations.* Implicit Association (IAT) (Greenwald et al., 1998) have been frequently used to assess changes in implicit racial bias (Farmer et al., 2012; Fini et al., 2013; Maister et al., 2013; Peck et al., 2013; Banakou et al., 2016) and gender bias (Lopez et al., 2019) after experiencing body swap illusions with an avatar from an outgroup. Banakou et al. (2016) argue that body perception influences implicit attitudes “*below the threshold of consciousness*” (p. 9) and although participants do not explicitly report that their bodies or attitudes changed, a change in the IAT score provides support for this relation. Therefore, similarly to Tacikowski et al. (2020a), we used an IAT pairing words describing *self, other, male*, and *female* to measure changes in implicit self-gender associations. The word stimuli were selected based on the gender IAT reported in Greenwald et al. (2002) and the order of the blocks was counterbalanced. The IAT was implemented in Qualtrics with the “iatgen” package (Carpenter et al., 2018) and participants pressed the keyboard keys “E” and “I” to sort stimuli to the left and right category respectively.

(c) *Baseline gender identity.* Besides self-categorisation measures such as the IAT, gender identity is often researched based on the association of an individual with stereotypical attributes or traits of males and females (Wood and Eagly, 2015). To provide richer insights into the gender identity of the sample, the Traditional Masculinity-Femininity (TMF) scale (Kachel et al., 2016) was administered which measures self-ascribed masculinity and femininity in relation to perceived gender roles and stereotypes (interests, attitudes, behaviour, and appearance). Thereby, the first two questions closely resemble the ones used by Tajadura-Jiménez et al. (2019), only asking “I would like to be…” instead of “I wish to be…” in the second question which allows for a better comparison with their sample. All questions are included in the Supplementary Methods.

(d) *Explicit self-gender group identification.* Identification with the group of women and the group of men was measured with a variation of the Inclusion of the Other in the Self (IOS) scale (Aron et al., 1992), one for each gender group respectively. The IOS is a pictorial measure of closeness. It consists of seven pictures, each displaying two circles of decreasing distance, and has been used for measuring identification with different social groups (Schubert and Otten, 2002), and also for assessing gender identification (Hundhammer and Mussweiler, 2012). In this experiment, one circle represents the Self and the other circle represents the group of women (IOS Women) or the group of men (IOS Men) respectively. Participants were asked to select the picture that represents their relationship to the respective group best. The closer the circles are to one another, the closer is the perceived relationship to the group.

(e) *Emotional state.* Similar to previous work (Tajadura-Jiménez et al., 2019), emotional state was measured with self-assessment manikins (Bradley and Lang, 1994) on a 9-point scale, respectively, for valence, arousal, and dominance. Based on these questions, it was assessed whether the emotional experience of the participants differed between the sound conditions, as for example increased arousal can further enhance the dominant IAT response (Gawronski and Bodenhausen, 2006).

(f) *Shape and weight concerns.* Previous research has shown that individuals with a (history of) eating disorders differ in their body perception and sensitivity to bodily illusions (Eshkevari et al., 2012, 2014). Not having any (history of) eating disorders was part of the selection criteria for the participants. However, to assess differences in this dimension, participants were asked to answer two subscales of the Eating Disorder Examination Questionnaire (EDE-Q) for weight and shape concerns (Fairburn and Beglin, 1994; Fairburn, 2008).

The Supplementary Methods contain an overview of all questions, answer options, and tasks which were used in this experiment.

#### Experimental Design

We used a within-subject design with two sound conditions: high and low frequency footstep sounds. Participants heard their own altered footstep sounds through headphones. Identical to previous research (Tajadura-Jiménez et al., 2015, 2019), in the *high frequency* condition, frequencies in the range of 1–4 kHz were amplified by 12 dB and the frequencies in the range of 83–250 Hz were attenuated by 12 dB. Conversely, in the *low frequency* condition, frequencies in the range of 83–250 Hz were amplified by 12 dB and frequencies in the range of 1–4 kHz were attenuated by 12 dB. Note that there was no walking condition without sound modification. As this study focussed on the malleability of gender identity within each participant in response to the altered footstep sounds, we included the two extremes (high frequency condition/low frequency condition) to explore potential changes. Each participant completed one high frequency condition and one low frequency condition walking phase. Sound order was counterbalanced across participants.

#### Procedure

After arriving in the lab, participants received written information about the experiment, were given the opportunity to ask any questions, and were then asked to sign an informed consent form. Participants completed a computerised version of the IAT for implicit self-gender association and then answered a set of questions (TMF, IOS Women, IOS Men). These tests provided a baseline control pre-intervention measurement. Then, six Notch movement sensors were attached to the participant’s body, and the participants put on the shoe prototype and backpack. The experimenter then attached one microphone each to the outside of the left and right sandal. Participants were then instructed to stand at the beginning of the wooden corridor and the Notch movement sensors were calibrated. Then, the experimenter gave the participant instructions for the walking phase and asked them to walk as if they would do normally. If there were no questions on the procedure the participants put on the headphones. After a visual starting signal, participants marched on the spot for 30 s and paused briefly after a visual stopping signal. Following a second visual starting signal, they walked down the corridor and paused at the end. The total exposure time to the altered footstep sounds was 35–40 s. Two separate recordings of movement data were collected in the Notch app, one for walking on the spot and one for walking down the corridor. After the walking was completed, participants took off the headphones, microphones, and backpack. They were then asked to sit down and complete the IAT task followed by IOS Women, IOS Men, bodily feelings, and SAM. This procedure was repeated with the second sound condition. There were approximately 8–12 min between the sound exposure in the two conditions. At the end of the experiment, participants answered additional questions on their weight and shape concern, thoughts on the purpose of the experiment, prior experience, and demographics. Finally, after taking off the Notch sensors and sandals, participants were asked about their body height and to step on a scale to measure body weight because previous work (Tajadura-Jiménez et al., 2019) identified body weight as a relevant factor for the effect of the sounds. All participants were fully debriefed. The total procedure took about 45 min.

#### Data Analyses

We report *p*-values smaller than 0.05 as significant. *P*-values in the range 0.05 to 0.1 are reported as marginally significant and corresponding trends are interpreted.

##### Body perception as quantified by bodily feelings and motor behaviour (H1)

**FIGURE 2:**
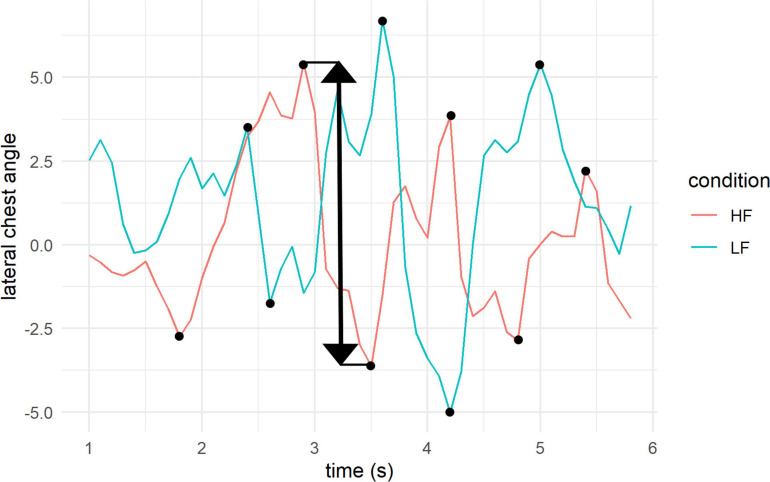
Example plot of lateral chest tilt in high and low frequency. The black dots represent the manually annotated peaks and valleys. We calculated the absolute differences between a consecutive peak and valley (black arrow). Based on these distances, two values were extracted: the average distance (sum divided by the total number of peaks and valleys) and the maximal distance between consecutive peaks and valleys in a walking phase.

Bodily feelings were evaluated using the three questions from the bodily feelings questionnaire. We compared the answers after walking in the high and low frequency conditions using non-parametric Wilcoxon signed-rank tests. To check for a potential effect of order of condition, the data was aligned rank transformed with the ARTool package (v. 0.10.6)^3^ in R, which allows a consecutive analysis with a two-way mixed ANOVA with order as a between-subjects factor. To assess potential effects on motor behaviour, we extracted CSV files with lateral hip and chest angles from the Notch recordings during the walking phase on the corridor. An automatic annotation of steps was tested but assessed to be unreliable due to high variability and noise within the data. Therefore, angles were plotted in MATLAB and peaks and valleys were manually annotated and extracted with the “data cursor mode” (see Figure 2). The total number of steps differed due to step size of the participants and some steps were overlaid by noise. Only those steps with a reoccurring, regular pattern were annotated. The manually annotated peaks and valleys were then used to extract from each walking phase: the average difference between a consecutive peak and valley (i.e., sum of distances between a consecutive peak and valley divided by total number of peaks and valleys) and the maximal difference between a consecutive peak and valley. The resulting data was not normally distributed; therefore, Wilcoxon signed-rank tests were used for statistical analysis. In the analysis, it became apparent that the results were strongly affected by the choice of metric (mean vs. maximal difference), included participants (with varying noise levels), and included number of steps. Given the high variability of signal-to-noise ratio between participants and pattern of statistical results depending on criteria, we decided to exclude the movement data from the reported analysis. Therefore, only the self-reported bodily feelings will be reported for H1.

##### Implicit self-gender association (IAT) (H2)

Implicit self-gender association was evaluated with the IAT, using the improved IAT scoring algorithm reported in Greenwald et al. (2003). A positive IAT score (0 to + 2] indicates an implicit self-female association; a negative IAT [−2 to 0) score indicates an implicit self-male association. After checking for normal distributions within each condition (Shapiro-Wilk test) and for sphericity (Mauchly’s Test for Sphericity), a repeated measures ANOVA was used to compare IAT scores between pre-test baseline, after high frequency, and after low frequency. *Post hoc* comparisons were done using Bonferroni corrected paired *t*-tests. Also, a two-way mixed ANOVA with order as a between-subjects factor was calculated to check for a potential effect of order.

##### Explicit self-gender group identification (IOS) (H3)

Explicit self-gender group identification was measured using a coded (1–7) version of the IOS scale. Increasing numbers corresponded to a closer proximity between the circles. For both the IOS with the group of men and the IOS with the group of women, a non-parametric Friedman test was calculated to compare the pre-test baseline measurement with the results after the high, and after the low frequency condition. *Post hoc* tests were done using non-parametric Wilcoxon signed-rank tests with Bonferroni correction. Analogous to the bodily feelings data, the IOS data was aligned rank transformed and analysed with a two-way mixed ANOVA to check for a potential effect of order.

##### Baseline gender identity

The answers to the TMF scale were coded with numbers from 1 to 7 and mean scores were calculated for all six questions (Kachel et al., 2016). As a benchmark, Kachel et al. (2016) reported mean TMF values of 4.54 (*SD* = 1.15) and 5.36 (*SD* = 0.72) for, respectively, lesbian and straight women. As sexual orientation was not assessed during this experiment, a *t*-test was calculated to compare our measured mean TMF score with the average of Kachel’s reported values for women (*M* = 4.95) to account for a potential diversity of sexual orientation in the sample. In addition, to allow a comparison with Tajadura-Jiménez et al. (2019), who only measured the first (masculine-feminine being) and second (masculine-feminine wish) question, we also analysed those questions individually. We compare our median scores to the medians reported in Tajadura-Jiménez et al. (2019).

##### Shape and weight concerns

We followed the coding instructions of Fairburn (2008), in which answers to the subscales are coded with numbers from 0 to 6. To allow a comparison of our observed scores with potential population scores, we compared the average shape and weight concern subscales with publicly available community norms for Australian undergraduate women (Mond et al., 2006).

### Results

Questionnaire data of one participant were excluded due to indicators of *content non-responsivity* (Meade and Craig, 2012) (i.e., giving the same answer to many consecutive questions, suggesting that question content was not read). Hence, the analysis of questionnaire data (H1 and H3) is based on 25 participants. Additional information on the female sample and comparisons to existing norms, for example for shape and weight concerns, are provided in the Supplementary Data S1. *Post hoc* correlations of implicit and explicit measures of gender identity are provided in the Supplementary Data S3.

#### Bodily Feelings (H1)

**FIGURE 3:**
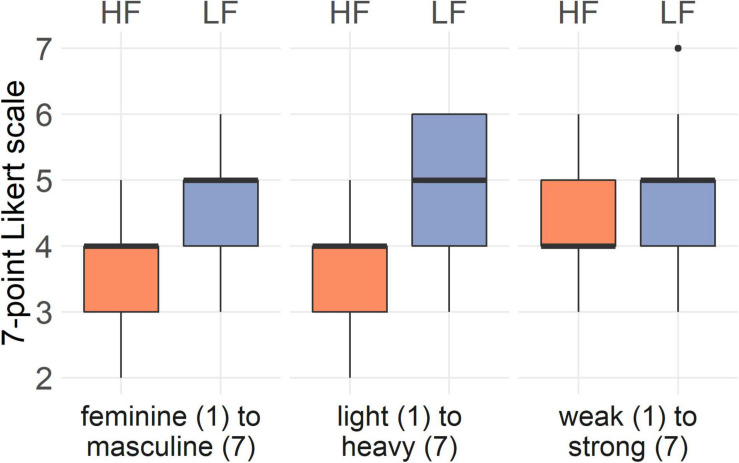
Answers of the women (*n* = 25) to the bodily feelings questions on perceived masculinity and femininity, body weight, and strength in the high frequency and low frequency condition. Black horizontal line shows median score; lower and upper hinge correspond to the first and third quartiles (25th and 75th percentiles).

After walking with high frequency step sounds, participants reported to feel significantly more feminine (*Z* = −3.46, *p* < 0.001, *r* = 0.69), lighter (*Z* = −3.43, *p* < 0.001, *r* = 0.69), and weaker (*Z* = −2.21, *p* = 0.027, *r* = 0.44) than after walking with low frequency step sounds. This trend is also reflected in the box-and-whisker plots in Figure 3. There was no significant interaction between the condition and the order of conditions for any of the bodily feelings (*p* > 0.05).

#### Implicit Self-Gender Association (IAT) (H2)

In the baseline measure, as expected, female participants associated themselves implicitly stronger with “female” than with “male” gender categories (IAT score: *M* = 0.38, *SD* = 0.4). The data was normally distributed within each point of measurement (Shapiro-Wilk test; baseline: *W* = 0.971, *p* = 0.651; high frequency: *W* = 0.982, *p* = 0.904; low frequency: *W* = 0.97, *p* = 0.631) and sphericity was not violated [χ^2^(2) = 0.386, *p* = 0.824]. Contrary to the formulated hypothesis, implicit self-gender association was not significantly affected by the walking sounds, *F*(2,50) = 0.366, *p* = 0.695, *n* = 26. There was also no significant interaction with the order of conditions [*F*(2,48) = 0.812, *p* = 0.448, *n* = 26)]. No further comparisons were calculated.

#### Explicit Self-Gender Group Identification (IOS) (H3)

**FIGURE 4:**
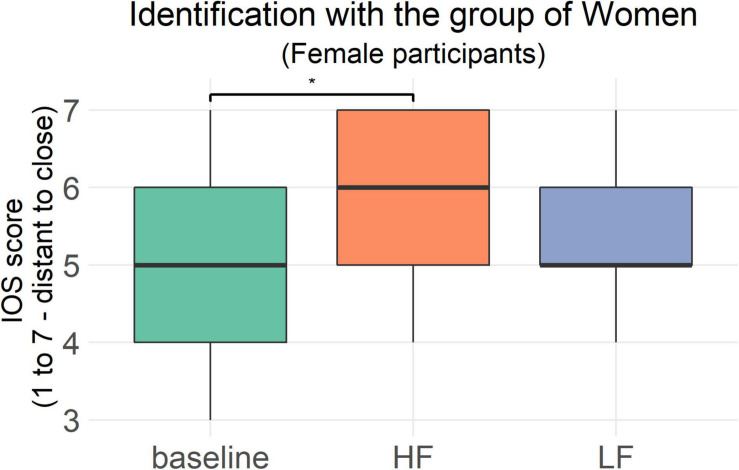
Answers of the women (*n* = 25) to the IOS scale for the group of women at the three points of measurement. The horizontal bar represents the median IOS score. A higher score indicates a closer relationship between the self and the group of women. *p ≤ 0.05.

In the baseline measure, as expected, women reported to feel significantly closer to the group of women (*M*_*women*_ = 5.32, *SD*_*women*_ = 1.15) compared to the group of men (*M*_*men*_ = 3.68, *SD*_*men*_ = 1.28; *Z* = −3.77, *p* < 0.001, *r* = 0.75). A higher mean indicates a closer proximity between the circles, that is a higher explicit self-gender group identification. As shown in Figure 4, there was a significant difference between the three points of measurement [baseline; after high frequency sounds; after low frequency sounds; χ^2^(2) = 8.18, *p* = 0.017, *n* = 25] with women reporting to feel closer to the group of women after walking with high frequency step sounds (*Z* = −2.47, *p*_*adjusted*_ = 0.04, *r_*adjusted*_* = 0.49) compared to the baseline.

The relationship to the group of men (IOS Men) did not differ between the three points of measurement [χ^2^(2) = 0.5, *p* = 0.78, *n* = 25]. Both for IOS Women and IOS Men there were no significant interactions between the condition and the order of conditions (*p* > 0.05). No further comparisons were calculated.

### Summary

Consistent with H1, the women in this experiment reported to feel lighter, more feminine, and weaker after walking with the high frequency step sounds compared to their perception after walking with low frequency step sounds. These findings align with previous work using altered footstep sounds (Tajadura-Jiménez et al., 2015, 2019) and confirm that the bodily illusion was induced successfully. The implicit self-association of women with “male” and “female” gender categories was not significantly affected by the altered footstep sounds (in contrast to H2). Consistent with H3, women indicated to feel closer to the group of women after walking with high frequency step sounds compared to the baseline measure. However, there were no differences in the reported identification with the group of men between the points of measurement.

## Experiment II: Altering Implicit Self-Gender Association and Explicit Self-Gender Group Identification of Men

### Motivation and Method

The first experiment supported the idea that altered footstep sounds can induce a gender illusion (as indicated by changes in perceived weight, femininity/masculinity, and strength) and possibly affect explicit self-gender group identification (IOS) of women. To investigate whether similar effects would occur for men, we conducted a second experiment with cisgender males. 26 cisgender males took part (*M* = 33.62 years, *SD* = 12.87 years). On average, they weighed 74.04 kg (*SD* = 10.09 kg) and were self-reportedly 178 cm (*SD* = 6.2 cm) tall. Their body mass index (*M* = 23.37, *SD* = 2.99, Range = 17.63 – 30.64) was in the healthy range (18.5 – 24.9) according to the National Health Service in the UK (see text footnote 1) for 18 participants. The eligibility criteria were identical to Experiment I. As men tend to have larger feet than women, we additionally included a required shoe size below UK 10 (EU 44) to ensure a good fit of the shoes. Participants were recruited through an online subject pool and the researcher’s social network. Each participant was compensated with £7 as an individual financial compensation.

Ethical approval was obtained by the UCL Research Ethics Committee (Project ID: UCLIC/1516/003/Staff). The study was performed according to institutional ethics and international standards for the protection of human participants. All participants provided their written informed consent prior to their participation and were fully debriefed.

The hypotheses were identical to those for Experiment I. All materials and procedures remained the same, only the order of the IOS scales was swapped, such that the male participants always answered the IOS for the group of men first. We also asked participants during debriefing whether they had noticed a difference between the sounds and – if so – how they would describe the difference. This was done to get more insight into participants’ experience, as informal chats with the participants from Experiment I revealed that participants differed in their interpretation of the variation across the sound conditions, for example perceiving differences in volume or noise level. Finally, we also asked participants whether English was their native language, since it was suspected that the command of the English language could affect IAT responses.

The analysis was adjusted to compare baseline gender identity, and shape and weight concerns with respective values for the group of men. Specifically, a *t*-test was calculated to compare the mean TMF scores with the average (*M* = 3) of the reported TMF means for straight men at 2.51 (*SD* = 0.98) and for gay men at 3.49 (*SD* = 0.87) by Kachel et al. (2016) to account for potential diversity of sexual orientation in the sample. The shape and weight concerns were compared to the respective norms from undergraduate men in the US (Lavender et al., 2010).

### Results

Additional information on the male sample and comparisons to existing norms, for example for shape and weight concerns, are provided in the Supplementary Data S2. *Post hoc* correlations of implicit and explicit measures of gender identity are provided in the Supplementary Data S3.

#### Bodily Feelings (H1)

**FIGURE 5:**
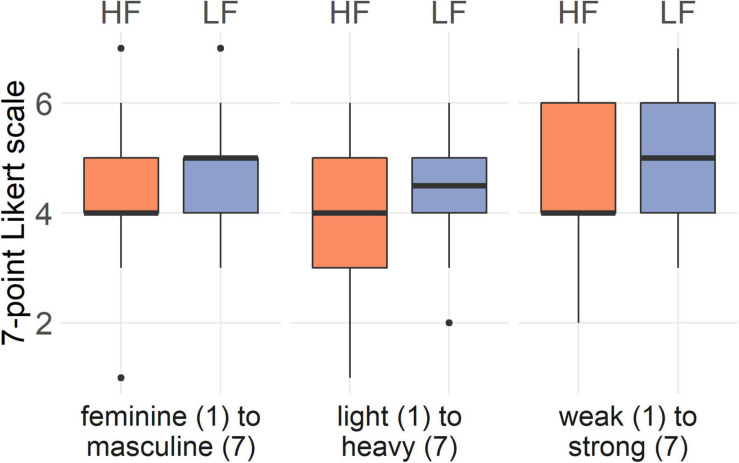
Answers of the men to the bodily feelings questions on perceived masculinity and femininity, body weight, and strength in the high frequency and low frequency condition. Black horizontal line shows median score; lower and upper hinge correspond to the first and third quartiles (25th and 75th percentiles).

After walking with high frequency step sounds, participants reported to feel significantly more feminine (*Z* = −2.03, *p* = 0.042, *r* = 0.4) than after walking with low frequency step sounds. There were no significant differences for light-heavy (*Z* = −1.31, *p* = 0.19, *r* = 0.26) or weak-strong (*Z* = −0.53, *p* = 0.6, *r* = 0.1) perception between high and low frequency step sounds (Figure 5). There was no significant interaction between the condition and the order of conditions for any of the bodily feelings (*p* > 0.05).

#### Implicit Self-Gender Association (IAT) (H2)

**FIGURE 6:**
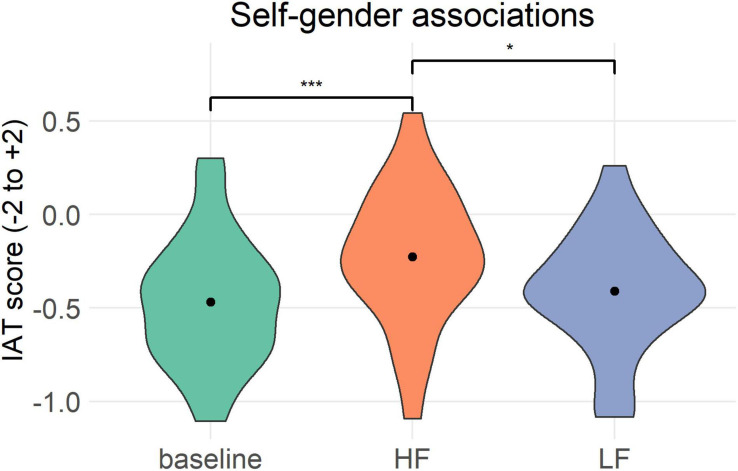
Distribution of IAT scores (–2 to + 2) in the group of men. The black dot indicates the mean IAT score. A positive score indicates quicker association of “Self” and “Female.” A negative IAT score indicates a quicker association of “Self” and “Male.” *p ≤ 0.05, ***p ≤ 0.001.

In the baseline measure, as expected, male participants associated themselves implicitly stronger with “male” than with “female” gender (IAT score: *M* = −0.47, *SD* = 0.34). The data was normally distributed within each point of measurement (Shapiro-Wilk test; baseline: *W* = 0.98, *p* = 0.87; high frequency: *W* = 0.987, *p* = 0.977; low frequency: *W* = 0.969, *p* = 0.604) and sphericity was not violated [χ^2^(2) = 0.759, *p* = 0.684]. The IAT scores differed significantly at the three time points [*F*(2,50) = 8.688, *p* < 0.001, η_*p*_^2^ = 0.258, *n* = 26]. Participants had significantly higher IAT scores after walking with the high frequency footstep sounds compared to the baseline [*t*(25) = −4.00, *p_*adjusted*_* < 0.001], and compared to the IAT score after walking with low frequency step sounds [*t*(25) = 3.02, *p_*adjusted*_* = 0.012]. Thus, participants implicitly associated themselves relatively less with “male” and more with “female” after walking with high frequency step sounds compared to both baseline and low frequency step sounds (Figure 6). There was no significant interaction between the condition and the order of conditions [*F*(2,48) = 0.587, *p* = 0.560, *n* = 26].

#### Explicit Self-Gender Group Identification (IOS) (H3)

In the baseline measures for explicit self-gender group identification (IOS), as expected, men reported closer group identification with the group of men (*M*_*men*_ = 4.96, *SD_*men*_* = 1.82) compared to the group of women (*M*_*women*_ = 3.5, *SD*_*women*_ = 1.61; *Z* = −2.42, *p* = 0.016, *r* = 0.47). A higher mean score represents a closer proximity between the circles, that is a closer explicit self-gender group identification. The group identification with the respective gender groups did not change in response to the altered footstep sounds, as there was no significant difference in the perceived closeness to the group of women [χ^2^(2) = 1.77, *p* = 0.412, *n* = 26] or the group of men [χ^2^(2) = 1.45, *p* = 0.485, *n* = 26] between the three points of measurement. Both for IOS Women and IOS Men there were no significant interactions between the condition and the order of conditions (*p* > 0.05).

We would have expected a change in explicit self-gender group identification (IOS) as the findings in the implicit self-gender associations (IAT) (H2) and the self-reported masculinity-femininity perception (H1) indicate a change in gender identity and this change is expected to affect explicit self-gender group identification (IOS) as well. Moreover, based on the mean IOS values, there was a tendency for men feeling closer to the group of women after walking in the low frequency condition compared to the other two conditions. As this is inconsistent with our hypothesis and the theory by Maister et al. (2015) and Tsakiris (2017), we examined the individual responses of the participants in more detail. Thereby, we noticed mismatches between the answers to the questions on perceived masculinity/femininity (TMF scale) and explicit self-gender group identification (IOS scales) for some of the participants (i.e., male participants 03 and 20; Supplementary Data). For example, male participant 20 indicated to be very masculine in the TMF scale [*M* = 1.33 on a scale from very masculine (1) to very feminine (7)] but then chose the most distant circles in the IOS for the group of men and the closest (i.e., completely overlapping) circles in the IOS for the group of women. One possible explanation for such mismatches could be that these participants misinterpreted the IOS scale to target *attraction toward* rather than *identification with* the respective gender group. Therefore, for some of the male participants, the IOS scale might have failed to capture the intended sense of belonging to the gender groups. For this reason, we decided not to interpret the IOS data from the second experiment further. We did not observe a similar pattern in the first experiment.

## Combined Analysis

Although previous work did not find differences in sound perception based on sex (Tajadura-Jiménez et al., 2019), the results from the two presented experiments suggest slight differences between the male and female participants. For the women in Experiment I, altered footstep sounds affected perceived masculinity and femininity, body weight, and strength as well as self-identification with the group of women, but not self-association with gender categories. For the men in Experiment II, however, altered footstep sounds affected self-association with gender categories and perceived masculinity and femininity, but not perceived body weight. Thus, an additional combined analysis was performed to explore potential interactions between the effect of the sound conditions and the sex of the participant.

### Combined Data Analyses

The ordinal data from the bodily feelings questions were aligned rank transformed with the ARTool package (v. 0.10.6) (see text footnote 3) in R, which allows a consecutive analysis with a two-way mixed ANOVA. The IAT data was not transformed and analysed with a two-way mixed ANOVA. Significant main effects were interpreted based on the interaction plots (see also Supplementary Figure 1) and for IAT data, a contrast analysis^4^ was performed with the emmeans package (v. 1.4.2)^5^ in R.

### Results

#### Bodily Feelings (H1)

**FIGURE 7:**
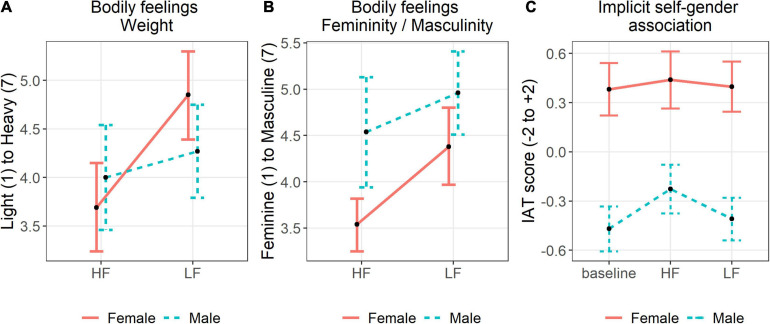
Visualisation of the data from male and female participants after walking with high and low frequency step sounds. **(A)** Shows mean values of perceived body weight (*n* = 51). **(B)** Shows mean values of perceived masculinity and femininity (*n* = 51). **(C)** shows mean IAT scores where a higher score corresponds to a stronger association with “Female” (*n* = 52). Error bars show 95% confidence intervals.

For the light-heavy perception, there was a significant main effect of sound condition [*F*(1,49) = 17.29, *p* < 0.001, η_*p*_^2^ = 0.261, *n* = 51], with participants feeling lighter after walking with the high frequency footstep sounds. The main effect of sex was not significant [*F*(1,49) = 0.002, *p* = 0.965, *n* = 51] but there was a significant interaction between sex and sound condition [*F*(1,49) = 5.98, *p* = 0.018, η_*p*_^2^ = 0.109, *n* = 51]. Visual inspection suggests that the change from high to low frequency was bigger for women than for men (Figure 7A).

For the masculine-feminine perception, there was a significant main effect of sound condition [*F*(1,49) = 25.48, *p* < 0.001, η_*p*_^2^ = 0.342, *n* = 51], with participants feeling more feminine after walking with the high frequency footstep sounds than with the low frequency step sounds. The main effect of sex was also significant [*F*(1,49) = 9.1, *p* = 0.004, η_*p*_^2^ = 0.157, *n* = 51] with women indicating to feel more feminine than men on average. The interaction between sex and sound condition was marginally significant [*F*(1,49) = 3.49, *p* = 0.068, η_*p*_^2^ = 0.067, *n* = 51]. Visual inspection suggests that the change from high to low frequency was bigger for women than for men (Figure 7B).

For the weak-strong perception, there was a significant main effect of sound condition [*F*(1,49) = 4.17, *p* = 0.047, η_*p*_^2^ = 0.078, *n* = 51], with participants tending to feel weaker after walking with the high frequency footstep sounds. Neither the main effect of sex [*F*(1,49) = 1.1, *p* = 0.3, *n* = 51] nor the interaction between sex and sound condition [*F*(1,49) = 1.74, *p* = 0.194, *n* = 51] was significant.

#### Implicit Self-Gender Association (IAT) (H2)

The requirements for the two-way mixed ANOVA were met as sphericity was not violated [χ^2^(2) = 0.291, *p* = 0.865], the variances were homogenous according to Levene’s test (baseline: *p* = 0.312; after high frequency: *p* = 0.312; after low frequency: *p* = 0.260), and the data was normally distributed within each condition (see Shapiro-Wilk in analysis of H2 in Exp. I and II). The main effect of sound condition was significant [*F*(2,100) = 5.744, *p* = 0.004, η_*p*_^2^ = 0.103, *n* = 52] and the contrast analysis and visual inspection of the combined data in Figure 7C revealed a significantly stronger implicit self-female association after walking with high frequency step sounds compared to the baseline measure (*t* = −3.08, *p_*adjusted*_* = 0.01, *n* = 52). The difference between the IAT scores after walking with high and with low frequency was significant as well (*t* = 2.49, *p*_*adjusted*_ = 0.048, *n* = 52). Both men and women implicitly associated themselves relatively less with “female” and more with “male” after walking with low frequency step sounds compared to high frequency step sounds. As expected, the main effect of sex was also significant [*F*(1,50) = 75.644, *p* < 0.001, η_*p*_^2^ = 0.602, *n* = 52], with women having stronger implicit self-female associations (higher IAT scores) than men. There was no significant interaction between sex and sound condition [*F*(2,100) = 2.21, *p* = 0.115, *n* = 52].

## Discussion

We studied the link between body perception and the self-concept through real-time alteration of footstep sounds. In two experiments, we replicated the finding that footstep sounds affect perceived masculinity and femininity during walking (Tajadura-Jiménez et al., 2019), suggesting that auditory feedback can induce a temporary gender illusion (H1). Further, men (Experiment II) experienced a temporary change in their self-concept as they associated themselves relatively more with “female” after walking with high frequency footstep sounds and relatively more with “male” after walking with low frequency footstep sounds (H2). This supports the theory that the self-concept is rooted in body perception and therefore malleable through bodily illusions (Maister et al., 2015; Tsakiris, 2017). Moreover, the results partially support the hypothesised connection between body perception and self-identification with social groups (Tsakiris, 2017), as women (Experiment I) reported to feel closer to the group of women after walking with high frequency footstep sounds (H3). We did not observe a “swap” in gender identity induced by the altered footstep sounds but a strengthened (Experiment I) or weakened (Experiment II) identification with one’s own sex. Thus, our findings suggest the malleability of gender identity in response to an auditory-induced bodily illusion.

The combined analysis further strengthens the support for H1 and H2, hence, that altered footstep sounds affect perceived body weight, masculinity-femininity, and strength, as well as implicit self-gender association (IAT) of both males and females. The combined analysis also revealed an interaction between the malleability of body weight perception and the sex of the participants, suggesting that the change in perceived body weight was stronger for women than for men. H3 is partially supported for the explicit self-gender group identification (IOS) with women feeling closer to the group of women after walking with high frequency footstep sounds. The changes in bodily feelings (perceived body weight, femininity/masculinity, strength) (H1) resemble and support earlier findings using the shoe prototype for altering footstep sounds to induce bodily illusions (Tajadura-Jiménez et al., 2015, 2019) and we thus expect that the bodily illusion was successfully induced similarly to previous experiments. The observed changes in implicit self-gender association (IAT) (H2) are consistent with the recent work from Tacikowski et al. (2020a) who showed that experiencing a body swap illusion with an avatar of a different sex causes temporary changes in the gender identity of the participants. We extend these findings by showing that subtle illusions of one’s own actual body induced through auditory feedback can cause such changes in one’s gender identity as well.

### Illusory Changes of One’s Own Actual Body Can Lead to Changes in the Self-Concept

Our findings contribute to the theoretical understanding of the connection between body perception and social cognition. Previous work showed that body swap illusions can affect implicit biases toward the embodied group (Maister et al., 2013; Peck et al., 2013), and the effect is theorised to occur through a change in the perception of one’s own body and one’s self-concept (Maister et al., 2015; Tsakiris, 2017). Specifically, it has been argued that attitudes and beliefs about the Self are linked to the representation of the body, and they will be adjusted in response to an altered body representation in order to maintain consistency between the Self and the body representation (Maister et al., 2015). Hence, body swap illusions are thought to first cause changes in the mental representation of one’s own body and one’s self-concept to incorporate more features of the embodied group, and thereby increase the identification with that group. The increased identification then causes a transfer of one’s positive self-evaluation to the embodied group, which becomes apparent in the change in implicit associations (Maister et al., 2015; Tsakiris, 2017).

Our work addresses a gap in the literature of providing direct evidence for changes in the self-concept following direct changes of one’s own actual body (i.e., own body illusions). There is some evidence from previous work that body swap illusions cause changes in the self-concept (Banakou et al., 2013; Tajadura-Jiménez et al., 2017a; Tacikowski et al., 2020a,b) but none of these showed these effects for own body illusions. Our findings support the hypothesised connection between body perception and the self-concept by demonstrating that subtle auditory-induced illusions of direct changes of one’s own actual body can lead to temporary changes in gender identity, as reflected in changes in implicit self-gender associations (IAT) and explicit self-gender group identification (IOS).

It is noteworthy that the IAT which was used for this experiment measured implicit self-gender association with word stimuli. Hence, the IAT assessed an abstract, semantic conceptualisation of the Self. The observed change in the IAT score therefore suggests that the bodily illusion did not only affect participants’ physical self-perception but also their higher-level conceptual Self. This is different from the majority of previous work on changes in the self-concept which assessed changes in implicit associations based on the sensory features that were manipulated during the bodily illusion. For example, Banakou et al. (2013) and Tajadura-Jiménez et al. (2017a) altered the physical appearance of participants by inducing a body ownership illusion of a child avatar in VR and used an IAT with *images* of adults and children to measure self-association with adults and children, hence, staying in the same sensory domain. Only the recent work by Tacikowski et al. (2020a) provides evidence for this transfer as they measured the effects of a body swap illusion with an IAT using semantic stimuli. We add to their work by demonstrating this effect for auditory-induced *own* body illusions.

Our findings also support the grounded or embodied approach to human cognition which assumes that our higher-level cognition is grounded in multimodal sensory experiences. In this approach, considerable attention is paid to physical sensations and the relationship between the body and the brain (Barsalou, 2008), exploring for example the influence of fluid movements on creativity (Slepian and Ambady, 2012). In the context of bodily illusions, previous research on body swap illusions found that embodying an Albert Einstein avatar can enhance performance in a cognitive task (Banakou et al., 2018) and that gender swap illusions in VR can improve performance in stereotype threatening situations [i.e., situations in which an individual’s performance is affected by a negative stereotype: that the group is expected to perform worse (Peck et al., 2018)]. Future work should investigate whether auditory-induced illusions of one’s own actual body can cause changes beyond the self-concept, for example alter one’s performance in stereotype threatening situations or one’s implicit attitudes toward others.

### Why Might Changes in Implicit Self-Gender Associations (IAT) Be Less Pronounced for Female Participants?

In Experiment I, while the implicit self-association (IAT) of women with male and female gender (H2) followed the predicted trend, the differences were not significant. As the combined analysis revealed a significant main effect of footstep sounds for all participants (without an interaction with gender; see Figure 7C), we suspect that the effect was not as pronounced in the first experiment due to characteristics of the tested female sample. We discuss a stronger focus on body weight and shape in the female sample as a possible explanation.

The frequency components in the footstep sounds are not only indicators for sex, but also for the body weight of the walker (Li et al., 1991), and have been shown to affect body weight perception in previous experiments (Tajadura-Jiménez et al., 2015, 2019). As the shape and weight concerns were higher among the females compared to males (see Supplementary Datas S1, S2), females might have interpreted the sounds more strongly in relation to body weight than in relation to sex. This explanation is consistent with the findings in the bodily feelings questions, where women self-reportedly experienced a change in perceived body weight while the effect was not significant for men (H1). Accordingly, the combined analysis suggested that the effect of sound condition on perceived body weight was stronger for females than for males. As previous research did not find differences between sexes in footstep sound perception (Tajadura-Jiménez et al., 2019), the found differences might be specific to the tested sample. Possibly, the social stigma associated with weight and gender may make some women more susceptible to the illusion (Tiggemann, 1994). However, this relation needs to be confirmed in future research.

### Implications for Research on the Malleability of Gender Identity

In addition to the implications for the connection between body and mind, our findings raise important questions about gender identity itself. While gender identity is generally considered to be stable for cisgender individuals, our experiments show that a short and subtle alteration of one’s body perception can temporarily affect gender identity. Gender identity is complex and while it does not have to be aligned with the appearance of one’s body, our results suggest that body-perception is at least an important facet in the perception of one’s gender identity.

Our results support the idea that gender identity should be understood as a continuum rather than distinct categories. The reason being that participants did not fully shift for example from identifying with male gender to identifying with female gender, but their gender identity became more balanced. These findings are in line with the work from Tacikowski et al. (2020a) who discuss the malleability of gender identity in response to bodily illusions in more detail. The results from the combined analysis which are relevant to gender identity showed no interactions with the participant’s sex and all trends (apart from the IOS Women scale) were identical in both experiments, which indicates that malleability of gender identity does not systematically differ between men and women.

### Limitations and Future Work

One limitation of our work is that we could not provide insights on whether an auditory-induced gender illusion is reflected in movement behaviour (H1) as, due to a poor signal-to-noise ratio, we decided not to interpret our Notch movement data further. Given that other auditory bodily illusions have affected movement patterns (Tajadura-Jiménez et al., 2016, 2019), and that males and females have different walking styles (Troje, 2002), we consider it likely that the walking patterns change in response to a gender illusion. The Notch sensors were calibrated in the laboratory with all electronic devices present, however, it is possible that the *activity* of the electronic devices during the experiment (e.g., the program for data collection, the active shoe prototype, setup for sound alteration, and devices from the participants) interfered with the signal from the Notch sensors. Therefore, in future research, further reducing signal interferences where possible and measuring more steps with the sensors would allow a more reliable identification of the consistent features (and distinction from the noisy features) in the signal of the steps.

A second limitation is that we cannot conclusively answer whether changes in explicit self-gender group identification (IOS) also occurred for men (H3) as, due to a suspected misinterpretation of the IOS scale by at least two of the male participants, we decided not to interpret our IOS data from Experiment II further. To improve future research on explicit self-gender group identification using the IOS scale, we would recommend clarifying the instructions further, for example by adding that the scale does not target attraction. In addition, it would be interesting to explore alternative or implicit measures for self-gender group identification. For example, one simple modification could be to use a continuous scale with two circles instead of multiple images, asking participants to manually adjust the distance between the circles to reflect their sense of belonging. This could capture a more fine-grained and intuitive sense of group identification and prevent the response artefact of participants reselecting their previous answer option without considering their immediate experience.

Third, several women (Experiment I) had heard of the shoe prototype and its connection to body weight prior to their participation (Supplementary Data S1). Thereby, none of the women had listened to or experienced the sound alteration before taking part. Further, the differences between the conditions are quite subtle especially if they are not played seamlessly after one another. Thus, having heard about the prototype did not imply that participants were able to correctly identify the high (“light”) and low (“heavy”) frequency conditions. This is evidenced by the feedback provided by men in Experiment II: only six participants described the difference between the sound conditions in terms of light- or heaviness. As gender identity had not been linked to the altered footstep sounds in prior experiments, we do not expect that participants were able to allocate the conditions correctly. However, in future experiments, we would ask participants to describe explicitly how they perceived the respective conditions to gain a better understanding of their interpretation of the sounds.

Lastly, while our findings are consistent with the predictive coding account, both bottom-up (sensorimotor) mechanisms and top-down (stereotypes triggered by sounds) mechanisms could be playing a role in the fluidity of gender associations in response to footstep sounds. Thus, future experimental set-ups should include an additional asynchronous control condition in which the sounds are disassociated from the steps to disambiguate these mechanisms more clearly.

## Conclusion

In sum, we showed that subtle illusions of direct changes of one’s own actual body can cause temporary changes in the self-concept, in this case one’s gender identity and relation to gender groups. Our findings support recent theories on the connection between body perception and social cognition (Maister et al., 2015; Tsakiris, 2017), and the notion that gender identity is at least partially rooted in physical experiences of one’s body and therefore temporarily malleable through bodily illusions (Tacikowski et al., 2020a). As many people wear headphones in their daily lives, auditory information could potentially be used to induce bodily illusions in real-life situations without requiring a complex VR environment. Given that (strength of) gender identification influences self-stereotyping (Cadinu and Galdi, 2012), such an application could potentially alleviate the performance decrease of some women in stereotype threatening situations. Also, altered footstep sounds could be used to enhance body-sex change illusions in VR (Tacikowski et al., 2020a) and provide an interesting tool for individuals with gender dysphoria to explore the effects of an altered body perception on their gender identity and well-being.

## Data Availability Statement

The original contributions presented in the study are included in the article/Supplementary Material, further inquiries can be directed to the corresponding authors.

## Ethics Statement

The studies involving human participants were reviewed and approved by the UCL Research Ethics Committee (Project ID: UCLIC/1516/003/Staff). The participants provided their written informed consent to participate in this study. Written informed consent was obtained from the individual(s) for the publication of any potentially identifiable images or data included in this article.

## Author Contributions

AT-J developed the shoe prototype used in the experiments. SC designed and conducted the experiment and wrote the first draft of the manuscript. SC and AT-J analysed the data. All authors discussed the experimental design and the results, and contributed to and approved the final manuscript.

## Conflict of Interest

The authors declare that the research was conducted in the absence of any commercial or financial relationships that could be construed as a potential conflict of interest.

## Publisher’s Note

All claims expressed in this article are solely those of the authors and do not necessarily represent those of their affiliated organizations, or those of the publisher, the editors and the reviewers. Any product that may be evaluated in this article, or claim that may be made by its manufacturer, is not guaranteed or endorsed by the publisher.

## Footnotes

1 NHS healthy weight. URL: https://www.nhs.uk/live-well/healthy-weight/

2 Notch Movement Sensors. URL: https://wearnotch.com

3 ARTool: Aligned Rank Transform. URL: https://cran.r-project.org/web/packages/ARTool/index.html

4 Contrast tests with ART. URL: https://cran.r-project.org/web/packages/ARTool/vignettes/art-contrasts.html

5 Emmeans: Estimated Marginal Means, aka Least-Squares Means. URL: https://cran.rproject.org/web/packages/emmeans/

## References

[B1] Apps M. A. J. Tsakiris M. (2014). The free-energy self: a predictive coding account of self-recognition. *Neurosci. Biobehav. Rev.* 41 85–97. 10.1016/j.neubiorev.2013.01.029 23416066 PMC3848896

[B2] Aron A. Aron E. N. Smollan D. (1992). Inclusion of other in the self scale and the structure of interpersonal closeness. *J. Pers. Soc. Psychol.* 63:596. 10.1037/0022-3514.63.4.596

[B3] Banakou D. Groten R. Slater M. (2013). Illusory ownership of a virtual child body causes overestimation of object sizes and implicit attitude changes. *Proc. Natl. Acad. Sci. U.S.A.* 110 12846–12851. 10.1073/pnas.1306779110 23858436 PMC3732927

[B4] Banakou D. Hanumanthu P. D. Slater M. (2016). Virtual embodiment of white people in a black virtual body leads to a sustained reduction in their implicit racial bias. *Front. Hum. Neurosci.* 10:601. 10.3389/fnhum.2016.00601 27965555 PMC5126081

[B5] Banakou D. Kishore S. Slater M. (2018). Virtually being Einstein results in an improvement in cognitive task performance and a decrease in age bias. *Front. Psychol.* 9:917. 10.3389/fpsyg.2018.00917 29942270 PMC6004376

[B6] Barsalou L. W. (2008). Grounded cognition. *Annu. Rev. Psychol.* 59 617–645. 17705682 10.1146/annurev.psych.59.103006.093639

[B7] Botvinick M. Cohen J. (1998). Rubber hands ‘feel’touch that eyes see. *Nature* 391:756. 10.1038/35784 9486643

[B8] Bradley M. M. Lang P. J. (1994). Measuring emotion: the self-assessment manikin and the semantic differential. *J. Behav. Ther. Exp. Psychiatry* 25 49–59. 10.1016/0005-7916(94)90063-9 7962581

[B9] Cadinu M. Galdi S. (2012). Gender differences in implicit gender self-categorization lead to stronger gender self-stereotyping by women than by men. *Eur. J. Soc. Psychol.* 42 546–551. 10.1002/ejsp.1881

[B10] Carpenter T. Pogacar R. Pullig C. Kouril M. Aguilar S. J. LaBouff J. P. (2018). Survey-based implicit association tests: a methodological and empirical analysis. *Behav. Res. Methods* 51 2194–2208. 10.3758/s13428-019-01293-3 31515742

[B11] Clark A. (2013). Whatever next? Predictive brains, situated agents, and the future of cognitive science. *Behav. Brain Sci.* 36 181–204. 10.1017/s0140525x12000477 23663408

[B12] Ehrsson H. H. (2012). “The concept of body ownership and its relation to multisensory integration,” in *The New Handbook of Multisensory Process*, ed. Stein B. E. (Cambridge, MA: MIT Press).

[B13] Eshkevari E. Rieger E. Longo M. R. Haggard P. Treasure J. (2012). Increased plasticity of the bodily self in eating disorders. *Psychol. Med.* 42 819–828. 10.1017/s0033291711002091 22017964

[B14] Eshkevari E. Rieger E. Longo M. R. Haggard P. Treasure J. (2014). Persistent body image disturbance following recovery from eating disorders. *Int. J. Eat. Disord.* 47 400–409. 10.1002/eat.22219 24243423

[B15] Fairburn C. G. (2008). *Cognitive Behavior Therapy and Eating Disorders.* New York, NY: Guilford Press.

[B16] Fairburn C. G. Beglin S. J. (1994). Assessment of eating disorders: interview or self-report questionnaire? *Int. J. Eat. Disord.* 16 363–370. 7866415

[B17] Farmer H. Maister L. Tsakiris M. (2014). Change my body, change my mind: the effects of illusory ownership of an outgroup hand on implicit attitudes toward that outgroup. *Front. Psychol.* 4:1016. 10.3389/fpsyg.2013.01016 24454301 PMC3888940

[B18] Farmer H. Tajadura-Jiménez A. Tsakiris M. (2012). Beyond the colour of my skin: how skin colour affects the sense of body-ownership. *Conscious. Cogn.* 21 1242–1256. 10.1016/j.concog.2012.04.011 22658684 PMC3772504

[B19] Fini C. Cardini F. Tajadura-Jiménez A. Serino A. Tsakiris M. (2013). Embodying an outgroup: the role of racial bias and the effect of multisensory processing in somatosensory remapping. *Front. Behav. Neurosci.* 7:165. 10.3389/fnbeh.2013.00165 24302900 PMC3831089

[B20] Gawronski B. Bodenhausen G. V. (2006). Associative and propositional processes in evaluation: an integrative review of implicit and explicit attitude change. *Psychol. Bull.* 132 692–731. 10.1037/0033-2909.132.5.692 16910748

[B21] Giordano B. L. Egermann H. Bresin R. (2014). The production and perception of emotionally expressive walking sounds: similarities between musical performance and everyday motor activity. *PLoS One* 9:e115587. 10.1371/journal.pone.0115587 25551392 PMC4281241

[B22] Greenwald A. G. Banaji M. R. Rudman L. A. Farnham S. D. Nosek B. A. Mellott D. S. (2002). A unified theory of implicit attitudes, stereotypes, self-esteem, and self-concept. *Psychol. Rev.* 109:3. 10.1037/0033-295x.109.1.3 11863040

[B23] Greenwald A. G. McGhee D. E. Schwartz J. L. K. (1998). Measuring individual differences in implicit cognition: the implicit association test. *J. Pers. Soc. Psychol.* 74 1464. 10.1037//0022-3514.74.6.1464 9654756

[B24] Greenwald A. G. Nosek B. A. Banaji M. R. (2003). Understanding and using the implicit association test: I. An improved scoring algorithm. *J. Pers. Soc. Psychol.* 85:197. 10.1037/0022-3514.85.2.197 12916565

[B25] Hundhammer T. Mussweiler T. (2012). How sex puts you in gendered shoes: sexuality-priming leads to gender-based self-perception and behavior. *J. Pers. Soc. Psychol.* 103:176. 10.1037/a0028121 22545746

[B26] Kachel S. Steffens M. C. Niedlich C. (2016). Traditional masculinity and femininity: validation of a new scale assessing gender roles. *Front. Psychol.* 7:956. 10.3389/fpsyg.2016.00956 27458394 PMC4932111

[B27] Lavender J. M. de Young K. P. Anderson D. A. (2010). Eating disorder examination questionnaire (EDE-Q): norms for undergraduate men. *Eat. Behav.* 11 119–121. 10.1016/j.eatbeh.2009.09.005 20188296

[B28] Li X. Logan R. J. Pastore R. E. (1991). Perception of acoustic source characteristics: walking sounds. *J. Acoust. Soc. Am.* 90 3036–3049. 10.1121/1.401778 1787243

[B29] Lopez S. Yang Y. Beltran K. Kim S. J. Cruz Hernandez J. Simran C. (2019). “Investigating implicit gender bias and embodiment of white males in virtual reality with full body visuomotor synchrony,” in *Proceedings of the 2019 CHI Conference on Human Factors in Computing Systems*, (Glasgow: ACM), 1–12.

[B30] Maister L. Sebanz N. Knoblich G. Tsakiris M. (2013). Experiencing ownership over a dark-skinned body reduces implicit racial bias. *Cognition* 128 170–178. 10.1016/j.cognition.2013.04.002 23680793 PMC3750641

[B31] Maister L. Slater M. Sanchez-Vives M. V. Tsakiris M. (2015). Changing bodies changes minds: owning another body affects social cognition. *Trends Cogn. Sci.* 19 6–12. 10.1016/j.tics.2014.11.001 25524273

[B32] Mather G. Murdoch L. (1994). Gender discrimination in biological motion displays based on dynamic cues. *Proc. R. Soc. B Biol. Sci.* 258 273–279. 10.1098/rspb.1994.0173

[B33] Meade A. W. Craig S. B. (2012). Identifying careless responses in survey data. *Psychol. Methods* 17:437. 10.1037/a0028085 22506584

[B34] Mond J. M. Hay P. J. Rodgers B. Owen C. (2006). Eating disorder examination questionnaire (EDE-Q): norms for young adult women. *Behav. Res. Ther.* 44 53–62. 10.1016/j.brat.2004.12.003 16301014

[B35] Paladino M.-P. Mazzurega M. Pavani F. Schubert T. W. (2010). Synchronous multisensory stimulation blurs self-other boundaries. *Psychol. Sci.* 21 1202–1207. 10.1177/0956797610379234 20679523

[B36] Peck T. C. Doan M. Bourne K. A. Good J. J. (2018). The effect of gender body-swap illusions on working memory and stereotype threat. *IEEE Trans. Visualiz. Comput. Graph.* 24 1604–1612. 10.1109/TVCG.2018.2793598 29543177

[B37] Peck T. C. Seinfeld S. Aglioti S. M. Slater M. (2013). Putting yourself in the skin of a black avatar reduces implicit racial bias. *Conscious. Cogn.* 22 779–787. 10.1016/j.concog.2013.04.016 23727712

[B38] Schubert T. W. Otten S. (2002). Overlap of self, ingroup, and outgroup: pictorial measures of self-categorization. *Self Identity* 1 353–376. 10.1080/152988602760328012

[B39] Senna I. Maravita A. Bolognini N. Parise C. V. (2014). The marble-hand illusion. *PLoS One* 9:e0091688. 10.1371/journal.pone.0091688 24621793 PMC3951417

[B40] Slepian M. L. Ambady N. (2012). Fluid movement and creativity. *J. Exp. Psychol. Gen.* 141:625. 10.1037/a0027395 22352395

[B41] Stanton T. R. Spence C. (2020). The influence of auditory cues on bodily and movement perception. *Front. Psychol.* 10:3001. 10.3389/fpsyg.2019.03001 32010030 PMC6978806

[B42] Tacikowski P. Fust J. Ehrsson H. H. (2020a). Fluidity of gender identity induced by illusory body-sex change. *Sci. Rep.* 10:14385. 10.1038/s41598-020-71467-z 32873869 PMC7463009

[B43] Tacikowski P. Weijs M. L. Ehrsson H. H. (2020b). Perception of our own body influences self-concept and self-incoherence impairs episodic memory. *Iscience* 23:101429. 10.1016/j.isci.2020.101429 32853552 PMC7520895

[B44] Tajadura-Jiménez A. Banakou D. Bianchi-Berthouze N. Slater M. (2017a). Embodiment in a child-like talking virtual body influences object size perception, self-identification, and subsequent real speaking. *Sci. Rep.* 7:9637. 10.1038/s41598-017-09497-3 PMC5575082 28851953

[B45] Tajadura-Jiménez A. Basia M. Deroy O. Fairhurst M. Marquardt N. Bianchi-Berthouze N. (2015). “As light as your footsteps: altering walking sounds to change perceived body weight, emotional state and gait,” in *Proceedings of the 33rd Annual ACM Conference on Human Factors in Computing Systems - CHI ‘15*, eds Begole B. Kim J. Inkpen K. Woo W. (New York, NY: ACM Press), 2943–2952.

[B46] Tajadura-Jiménez A. Deroy O. Marquardt T. Bianchi-Berthouze N. Asai T. Kimura T. (2018). Audio-tactile cues from an object’s fall change estimates of one’s body height. *PLoS One* 13:e0199354. 10.1371/journal.pone.0199354 29949607 PMC6021069

[B47] Tajadura-Jiménez A. Marquardt T. Swapp D. Kitagawa N. Bianchi-Berthouze N. (2016). Action sounds modulate arm reaching movements. *Front. Psychol.* 7:1391. 10.3389/fpsyg.2016.01391 27695430 PMC5025518

[B48] Tajadura-Jiménez A. Newbold J. Zhang L. Rick P. Bianchi-Berthouze N. (2019). “As light as you aspire to be: changing body perception with sound to support physical activity,” in *Proceedings of the 2019 CHI Conference on Human Factors in Computing Systems*, (Glasgow: ACM), 1–14.

[B49] Tajadura-Jiménez A. Vakali M. Fairhurst M. T. Mandrigin A. Bianchi-Berthouze N. Deroy O. (2017b). Contingent sounds change the mental representation of one’s finger length. *Sci. Rep.* 7:5748. 10.1038/s41598-017-05870-4 PMC5515978 28720803

[B50] Tiggemann M. (1994). Gender differences in the interrelationships between weight dissatisfaction, restraint, and self-esteem. *Sex Roles* 30 319–330. 10.1007/BF01420596

[B51] Troje N. F. (2002). Decomposing biological motion: a framework for analysis and synthesis of human gait patterns. *J. Vis.* 2:2. 10.1167/2.5.2 12678652

[B52] Tsakiris M. (2010). My body in the brain: a neurocognitive model of body-ownership. *Neuropsychologia* 48 703–712. 10.1016/j.neuropsychologia.2009.09.034 19819247

[B53] Tsakiris M. (2017). The multisensory basis of the self: from body to identity to others. *Q. J. Exp. Psychol.* 70 597–609. 10.1080/17470218.2016.1181768 27100132 PMC5214748

[B54] Wendy W. Eagly A. H. (2009). “Gender identity,” in *Handbook of Individual Differences in Social Behavior* 109–125.

[B55] Wood W. Eagly A. H. (2015). Two traditions of research on gender identity. *Sex Roles* 73 461–473. 10.1007/s11199-015-0480-2

