# Supplementary material for: Action Sounds Informing Own Body Perception Influence Gender Identity and Social Cognition
Source: Front Hum Neurosci. 2021 Jul 28;15:688170. doi: 10.3389/fnhum.2021.688170 (PMC8355547; doi:10.3389/fnhum.2021.688170)
Supplement: Supplementary file 1 [file Data_Sheet_1.docx]

Supplementary Material

# Supplementary Data S1: Experiment Women

The following section provides more information on the female sample (*n* = 26) to estimate the generalisability of the findings.

*Baseline gender identity.* The mean TMF score was 4.92 (*SD* = 0.66) and did not differ from the mean of the scores for lesbian and straight women (*t*(25) = -0.266, *p* = .793) reported by Kachel et al. (2016). This indicates that the baseline gender identity of the women in this experiment is comparable to the baseline gender identity of other women. Further, the median scores for masculine-feminine being (*Mdn* = 5) and masculine-feminine wish (*Mdn* = 5) were identical to the medians for women reported by Tajadura-Jiménez et al. (2019) suggesting that the women in both samples were similar in this regard.

*Shape and weight concern.* The average shape concern in the sample was 2.26 (*SD* = 1.71) and the weight concern was 2.03 (*SD* = 1.71). These concerns did not differ significantly from the Australian community norms (Mond et al., 2006) for shape concerns (*t*(25) = 0.087, *p*= .931) or weight concerns (*t*(25) = 0.718, *p* = .479) of young women.

*Prior knowledge.* 11 participants reported that they had completed an IAT before taking part in this experiment. 14 participants had heard of the shoe prototype before. Of those, 10 reported to know that the shoes could change body weight perception.

*Emotions.* There were no significant differences in emotional valence (Z = -1.22, *p* = .223, *r*= .24), arousal (Z = 0, *p* = 1, *r* = 0), or dominance (Z = -1.47, *p* = .141, *r* = .289) between the conditions, indicating that people experienced the two walking phases similarly.

# Supplementary Data S2: Experiment Men

The following section provides more information on the male sample (*n* = 26) to estimate the generalisability of the findings.

*Baseline gender identity.* The mean TMF score for male participants was 2.67 (*SD* = 1.1) and did not differ from the mean of the two scores for gay and straight men (*t*(25) = -1.55, *p* = .135) reported by Kachel et al. (Kachel et al., 2016), indicating that the baseline gender identity of the men in this experiment was comparable to the baseline gender identity of other men. The median scores for masculine-feminine being (*Mdn =*2.5) and for masculine-feminine wish (*Mdn* = 2) were slightly higher than the respective medians for the male participants in the sample of Tajadura-Jiménez et al. (2019).

*Shape and weight concern.* The mean shape concern in the male sample was 1.43 (*SD* = 1.2) and the mean weight concern was 1.36 (*SD =*1.3). These concerns did not differ significantly from the US community norms for shape concerns (*t*(25) = -0.67, *p*= .508) or weight concerns (*t*(25) = 0.28, *p* = .781) of young men (Lavender et al., 2010). Therefore, the body image of the men who participated in this experiment corresponded to existing norms.

*Prior knowledge.* 11 participants reported that they had completed an IAT prior to taking part in the experiment. 2 participants had heard of the shoe prototype before but neither of these knew that they could be used to changes body weight perception.

*Emotions*. There were no significant differences in valence (Z = -0.638, *p* = .524, *r* = .125), arousal (Z = -0.936, *p*= .349, *r* = .184), and dominance (Z = -0.218, *p =* .828, *r* = .043) between the low and high frequency step sounds.

*Topic.* 10 participants related the footstep sounds to perceived masculinity, femininity, or gender identity. 2 participants included the perception of other individuals in their explanation.

*Sound perception.* 20 participants reported to have noticed a difference between the sound conditions. Of these, 6 participants described the difference in terms of light or heavy sounds. Other descriptions referred to the type of shoes (*e.g.*, boots), the material of the ground (*e.g.*, wood), other features of the sounds (*e.g.*, sharpness) or emotional connotations (*e.g.*, calm).

# Supplementary Data S3: Correlations

While the relation between explicit and implicit measures of gender identity is still a matter of debate (Wood and Eagly, 2015) and implicit and explicit measures do not always align (Nosek, 2007), we explored these relations post hoc by calculating spearman correlations for the IAT and IOS/femininity-masculinity ratings. As for the analyses of H1 and H3 in Experiment I, all correlations are based on 25 participants. For Experiment II, 26 participants were included. We did not include the correlations with the IOS data from the second Experiment, as we decided not to analyse this data further. Scatter plots including a regression line were created for the significant and marginally significant correlations with the stat_cor function of the ggpubr 0.4.0 package^[[1]](#footnote-1)^ in R (Supplementary Figures 2-4).

Experiment I (*marginally significant; **significant)

- Correlation between IAT scores and femininity / masculinity ratings (n = 25)
  - After high frequency: rs = -0.26, *p* = .22
  - After low frequency: rs = -0.39, *p* = .055*
- Correlation between IAT scores and IOS for the group of Women scores (n = 25)
  - Baseline: rs = 0.42, p = .031**
  - After high frequency: rs = 0.24, *p* = 0.24
  - After low frequency: rs = 0.44, *p* = .026**
- Correlation between IAT scores and IOS for the group of Men scores (n = 25)
  - Baseline: rs = 0.07, *p* = .73
  - After high frequency: rs = -0.06, *p* = 0.77
  - After low frequency: rs = 0.25, *p* = .22

Experiment II:

- Correlation between IAT scores and femininity / masculinity ratings (n = 26)
  - After high frequency: rs = -0.13, *p* = .54
  - After low frequency: rs = -0.04, *p* = .85

The inspection of the scatter plots for the marginally significant and significant correlations (Supplementary Figures 2-4) showed that the pairs of measures relate to one another in the expected way. For the pair IAT - femininity / masculinity (Supplementary Figure 2), a lower IAT score (i.e., a stronger implicit self-male association (IAT) or a weaker implicit self-female association (IAT)) corresponds to a higher rating on the bodily feelings scale for femininity/masculinity (i.e., feeling more masculine). This correlation is marginally significant for the female group (Experiment I) after walking with the low frequency step sounds (rs = -0.39, *p* = .055).

For the pair IAT and IOS for the group of Women in Experiment I (Supplementary Figure 3 and 4), the correlation plot shows the expected trend: a lower IAT score (i.e., a stronger implicit self-male association (IAT) or a weaker implicit self-female association (IAT)) corresponds to a lower IOS women score (i.e., a weaker explicit self-gender group identification with the group of women). The correlations are significant at baseline (rs = 0.42, *p* = .031) and after low frequency sounds (rs = 0.44, *p* = .026).

# Supplementary Figures

#
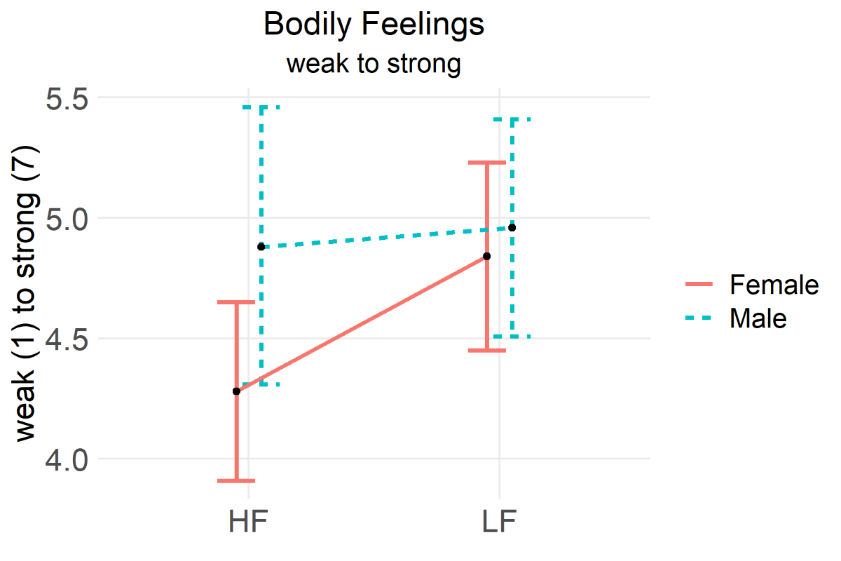


**Supplementary Figure 1.** Answers of females (Exp. I; *n* = 25), and males (Exp. II, *n*  = 26) to the bodily feelings question on perceived strength after walking with high and low frequency step sounds. Black dots are mean values and error bars indicate 95% confidence intervals.


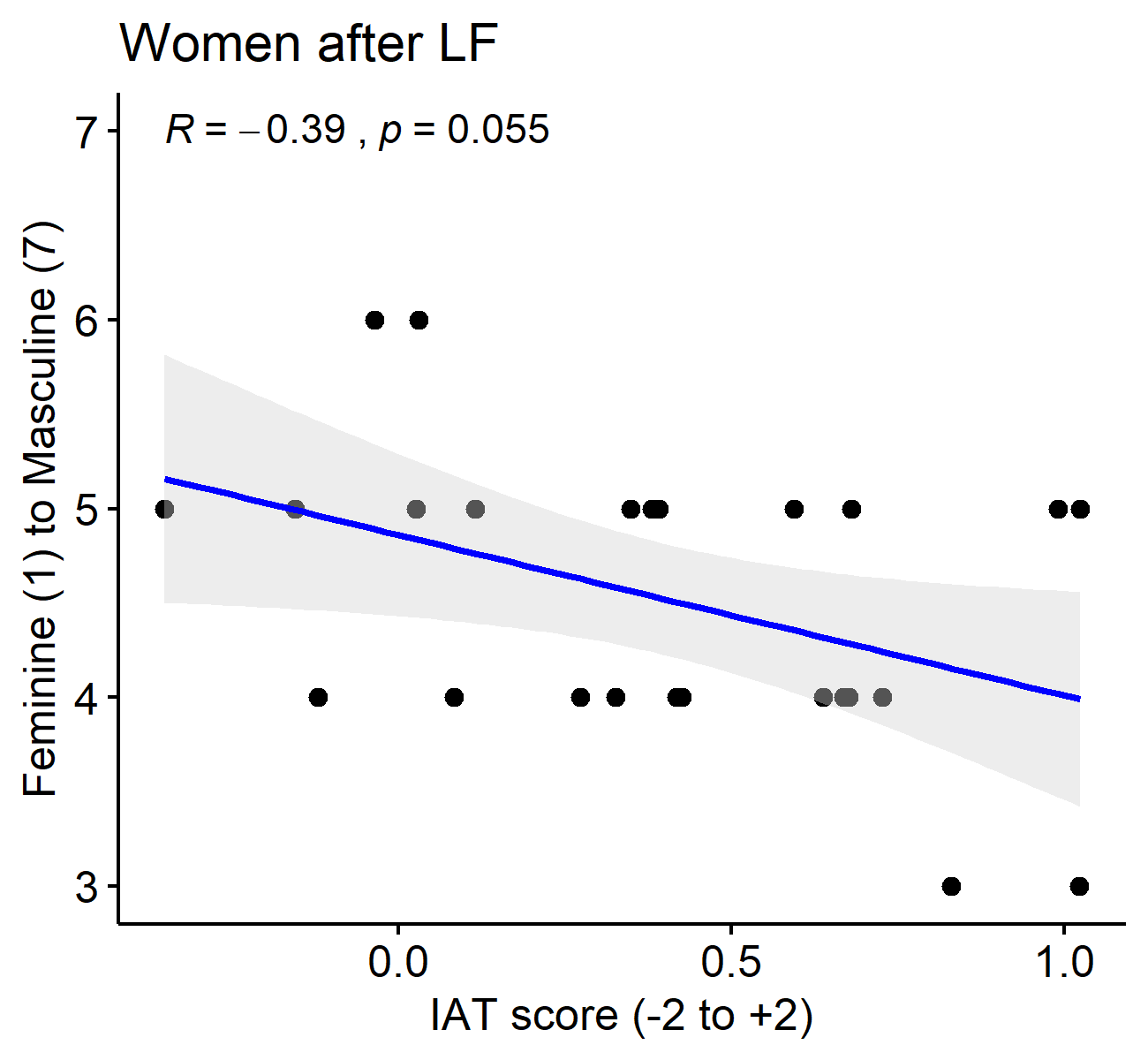


**Supplementary Figure 2.** Spearman correlation of implicit self-gender association (IAT) and perceived masculinity / femininity of females (Exp. I; *n* = 25) after the low frequency condition. In the plot, “*R*” corresponds to spearman’s rho.


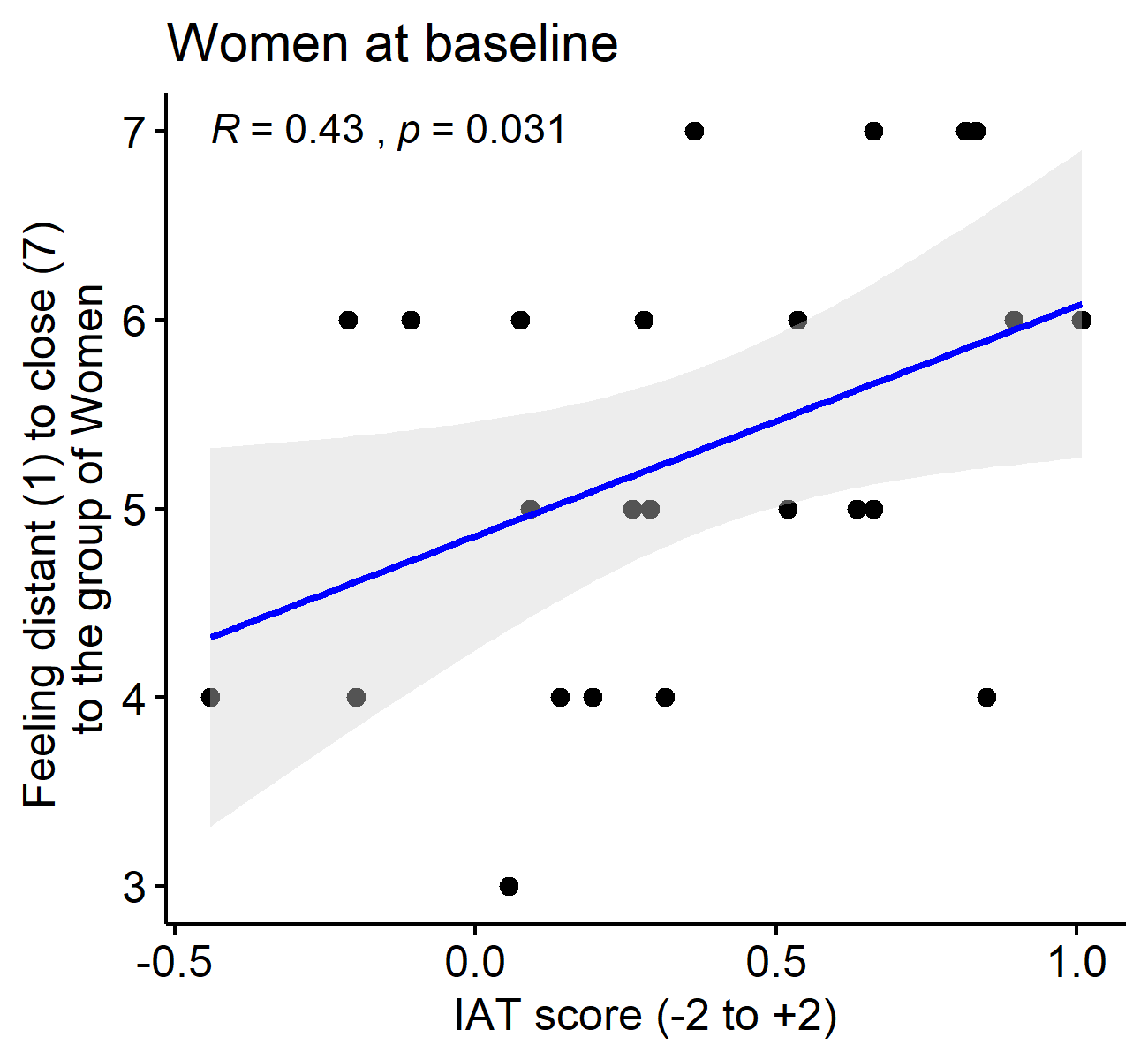


**Supplementary Figure 3.** Spearman correlation of implicit self-gender association (IAT) and explicit self-gender group identification with the group of Women (IOS) of females (Exp. I; *n* = 25) at the baseline measure. In the plot, “*R*” corresponds to spearman’s rho.


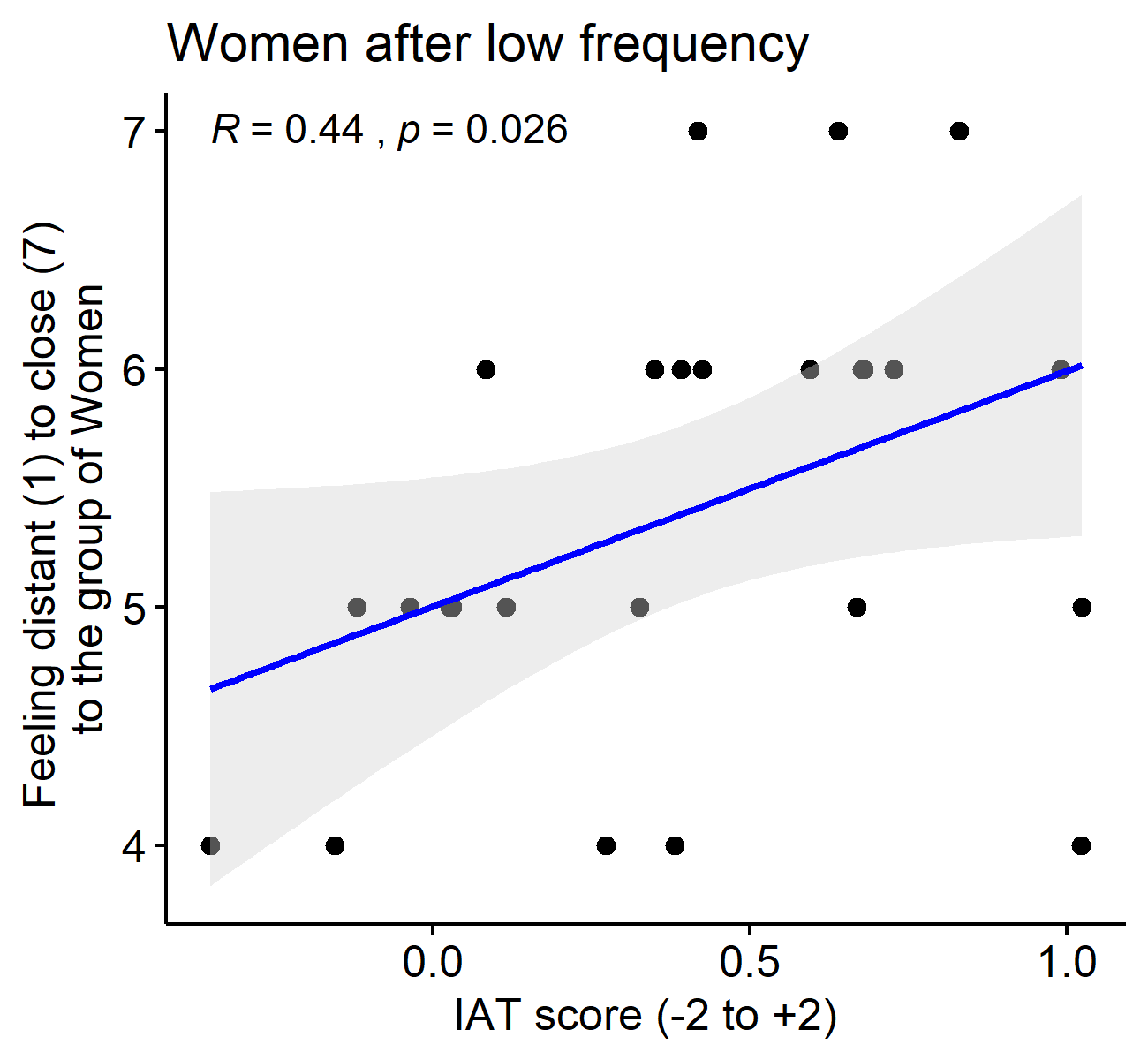


**Supplementary Figure 4.** Spearman correlation of implicit self-gender association (IAT) and explicit self-gender group identification with the group of Women (IOS) of females (Exp. I; *n* = 25) after walking with low frequency step sounds.

# **Supplementary Methods:** Full procedure incl. questions

| **Question ID** | **Variable Name** | **Question** | **Scoring Scale** |
| --- | --- | --- | --- |
| **Q1** | *Gender self-categorisation IAT (stimuli selected based on gender IAT in Greenwald et al. (2002))* | | |
|  | IAT_base | Self: I, me, mine, my, self  Other: them, their, theirs, they, other  Female: woman, she, her, girl, female  Male: man, he, him, boy, male | Calculation of d-score (-2 to +2) according to the improved scoring algorithm (Greenwald et al., 2003) |
| **Q2** | *Traditional Masculinity-Femininity Scale (TMF; Kachel et al. (2016)), calculation of MF_total* | | |
|  | TMF_1 / MF_being | ‘I consider myself to be…’ | 1 (Very masculine) to 7 (Very feminine) |
|  | TMF_2 / MF_wish | ‘I would like to be…’ |  |
|  | TMF_3 | ‘Traditionally, my interests would be considered as…’ |  |
|  | TMF_4 | ‘Traditionally, my attitudes and beliefs would be considered as…’ |  |
|  | TMF_5 | ‘Traditionally, my behaviour would be considered as…’ |  |
|  | TMF_6 | ‘Traditionally, my outer appearance would be considered as…’ |  |
| **Q3** | *Version of the Inclusion of the Other in the Self scale; Aron et al. (1992) to measure the overlap between the Self and in- and outgroup (Figure 1B in Schubert and Otten (2002))* | | |
|  | IOS_W_1* | ‘Choose the image that represents your relationship to the group of **women**’ | 1 (distant circles) to 7 (completely overlapping circles) |
| **Q4** | IOS_M_1* | ‘Choose the image that represents your relationship to the group of **men**’ | 1 (distant circles) to 7 (completely overlapping circles) |
| **Walking phase I** | | | |
| **Q5** | IAT_  postHigh or  postLow | Same stimuli as in Q1 | Same calculation of d-score as in Q1 |
| **Q6** | IOS_W_2* | ‘Choose the image that represents your relationship to the group of **women**’ | 1 (distant circles) to 7 (completely overlapping circles) |
| **Q7** | IOS_M_2* | ‘Choose the image that represents your relationship to the group of **men**’ | 1 (distant circles) to 7 (completely overlapping circles) |
| **Q8** | *Bodily feelings questions selected from Tajadura-Jiménez et al. (2019)* | | |
|  | LiHea_1 | ‘During the experience, I felt…’ | 1 (Light) to 7 (Heavy) |
|  | WeStr_1 | ‘During the experience, I felt…’ | 1 (Weak) to 7 (Strong) |
|  | FemMas_1 | ‘During the experience, I felt…’ | 1 (Very feminine) to 7 (Very masculine) |
| **Q9** | *Self-Assessment Manikins SAM (Bradley and Lang, 1994)* | | |
|  | SAM_Va_1 | Please look at the images above. Choose the image corresponding to how **happy** you feel. | Five images with numbers from 1 to 9 |
|  | SAM_Ar_1 | Please look at the images above. Choose the image corresponding to how **aroused** you feel. |  |
|  | SAM_Do_1 | Please look at the images above. Choose the image corresponding to how **dominant** you feel. |  |
| **Walking phase II** | | | |
| **Q10** | IAT_ postHigh or  postLow | Same stimuli as in Q1 | Calculation of d-score as in Q1 |
| **Q11** | IOS_W_3* | ‘Choose the image that represents your relationship to the group of **women**’ | 1 (distant circles) to 7 (completely overlapping circles) |
| **Q12** | IOS_M_3* | ‘Choose the graphic that image your relationship to the group of **men**’ | 1 (distant circles) to 7 (completely overlapping circles) |
| **Q13** | LiHea_2 | ‘During the experience, I felt…’ | 1 (Light) to 7 (Heavy) |
|  | WeStr_2 | ‘During the experience, I felt…’ | 1 (Weak) to 7 (Strong) |
|  | FemMas_2 | ‘During the experience, I felt…’ | 1 (Very feminine) to 7 (Very masculine) |
| **Q14** | SAM_Va_2 | ‘Please look at the images above. Choose the image corresponding to how **happy** you feel.’ | Five images with numbers from 1 to 9 |
|  | SAM_Ar_2 | ‘Please look at the images above. Choose the image corresponding to how **aroused** you feel.’ |  |
|  | SAM_Do_2 | ‘Please look at the images above. Choose the image corresponding to how **dominant** you feel.’ |  |
| **Q15** | *Shape and weight concern subscales from Eating Disorder Examination Questionnaire (EDE-Q (Fairburn and Beglin, 1994)); subscales coded according to Fairburn (2008) for the calculation of the variables ‘shape_concern’ and ‘weight_concern’* | | |
|  | ShaWei_1  ShaWei_2  ShaWei_3  ShaWei_4  ShaWei_5 | On how many of the past 28 days...  ‘Have you had a definite desire to have a totally flat stomach?’  ‘Has thinking about shape or weight made it very difficult to concentrate on things you are interested in (for example, working, following a conversation, or reading)?’  ‘Have you had a definite fear that you might gain weight?’  ‘Have you felt fat?’  ‘Have you had a strong desire to lose weight?’ | 0 (No days)  1 (1-5 days)  2 (6-12 days)  3 (13-15 days)  4 (16-22 days)  5 (23-27 days)  6 (All days) |
| **Q16** | ShaWei_2_1  ShaWei_2_2  ShaWei_2_3  ShaWei_2_4  ShaWei_2_5  ShaWei_2_6  ShaWei_2_7 | Over the past 28 days...  ‘Has your weight influenced how you think about (judge) yourself as a person?’  ‘Has your shape influenced how you think about (judge) yourself as a person?’  ‘How much would it have upset you if you had been asked to weigh yourself in front of others once a week for the next four weeks?’  ‘How dissatisfied have you been with your weight?’  ‘How dissatisfied you have been with your shape?’  ‘How uncomfortable have you felt seeing your body (for example, seeing your shape in the mirror, in a shop window reflection, while undressing or taking a bath or shower)?’  ‘How uncomfortable have you felt about others seeing your shape or figure (for example, in communal changing rooms, when swimming, or wearing tight clothes)?’ | 0 (Not at all)  1  2 (Slightly)  3  4 (Moderately)  5  6 (Markedly) |
| **Q17** | Topic | ‘What do you think this experiment was about?’ | Free text |
| **Q18** | PriorIAT | ‘Did you complete a word-sorting task similar to the first task in this survey (an Implicit Association Task or IAT) before taking part in this experiment?’ | 1 (Yes) / 0 (No) |
| **Q19** | PriorShoes  PriorShoes_2 | ‘Did you hear about the Magic Shoes before?’  If yes:  ‘Did you know prior to the experiment that the sounds can change your body weight perception?’ | 1 (Yes) / 0 (No)  1 (Yes) / 0 (No) |
| **Q20** | Gender | ‘Please choose your gender:’ | 1 (Female),  2 (Male),  3 (Non-binary),  4 (Prefer not to say),  Free text (Prefer to self-describe) |
| **Q21** | Age | ‘Please enter your age:’ | Free text |
| **Q22** | Nationality | ‘What is your nationality?’ | Free text |
| **Q23** | Height  Weight | Participants were asked for their height after completion of the survey; weight was measured with a scale |  |
| *Additional questions only asked in Experiment II (Males)* | | | |
| **Q23** | Sound_diff  Sound_description | ‘Did you hear a difference between the walking sounds?’  If yes:  ‘How would you describe the difference you heard?’ | 1 (Yes) / 0 (No)  Free text |
| **Q24** | Language | ‘Is English your native language?’ | 1 (Yes) / 0 (No) |

References

Aron, A., Aron, E. N., and Smollan, D. (1992). Inclusion of other in the self scale and the structure of interpersonal closeness. *Journal of personality and social psychology* 63, 596.

Bradley, M. M., and Lang, P. J. (1994). Measuring emotion: the self-assessment manikin and the semantic differential. *Journal of Behavior Therapy and Experimental Psychiatry* 25, 49–59.

Fairburn, C. G. (2008). *Cognitive behavior therapy and eating disorders*. Guilford Press.

Fairburn, C. G., and Beglin, S. J. (1994). Assessment of eating disorders: Interview or self‐report questionnaire? *International journal of eating disorders* 16, 363–370.

Greenwald, A. G., Banaji, M. R., Rudman, L. A., Farnham, S. D., Nosek, B. A., and Mellott, D. S. (2002). A unified theory of implicit attitudes, stereotypes, self-esteem, and self-concept. *Psychological review* 109, 3.

Greenwald, A. G., Nosek, B. A., and Banaji, M. R. (2003). Understanding and using the implicit association test: I. An improved scoring algorithm. *Journal of personality and social psychology* 85, 197.

Kachel, S., Steffens, M. C., and Niedlich, C. (2016). Traditional masculinity and femininity: Validation of a new scale assessing gender roles. *Front Psychol* 7, 956.

Lavender, J. M., Young, K. P. de, and Anderson, D. A. (2010). Eating Disorder Examination Questionnaire (EDE-Q): norms for undergraduate men. *Eating behaviors* 11, 119–121.

Mond, J. M., Hay, P. J., Rodgers, B., and Owen, C. (2006). Eating Disorder Examination Questionnaire (EDE-Q): Norms for young adult women. *Behaviour research and therapy* 44, 53–62. doi: 10.1016/j.brat.2004.12.003

Nosek, B. A. (2007). Implicit–explicit relations. *Current Directions in Psychological Science* 16, 65–69.

Schubert, T. W., and Otten, S. (2002). Overlap of self, ingroup, and outgroup: Pictorial measures of self-categorization. *Self and identity* 1, 353–376.

Tajadura-Jiménez, A., Newbold, J., Zhang, L., Rick, P., and Bianchi-Berthouze, N. (2019). “As Light as You Aspire to Be: Changing Body Perception with Sound to Support Physical Activity,” in *Proceedings of the 2019 CHI Conference on Human Factors in Computing Systems* (Glasgow, Scotland UK: ACM), 1-14.

Wood, W., and Eagly, A. H. (2015). Two Traditions of Research on Gender Identity. *Sex Roles* 73, 461–473. doi: 10.1007/s11199-015-0480-2

1. https://rpkgs.datanovia.com/ggpubr/reference/stat_cor.html [↑](#footnote-ref-1)
